# Efficacy and safety of spleen-strengthening and phlegm-resolving herbal therapy for residual dizziness after repositioning of benign paroxysmal positional vertigo: a systematic review and meta-analysis of randomized controlled trials

**DOI:** 10.3389/fmed.2026.1949948

**Published:** 2026-10-19

**Authors:** Yuqi Zhang, Yingqiu Sun, Qingxiao Li, Quanzhi Li, Yawei Du, Bingrui Zhang, Shengxian Wu

**Affiliations:** Dongzhimen Hospital, Beijing University of Chinese Medicine, Beijing, China

**Keywords:** benign paroxysmal positional vertigo, meta-analysis, residual dizziness, spleen-strengthening and phlegm-resolving, traditional Chinese medicine

## Abstract

**Introduction:**

Spleen-strengthening and phlegm-resolving (SSPR) herbal therapy, a classical core approach in traditional Chinese medicine (TCM) has been widely applied to treat residual dizziness (RD) after canalith repositioning procedures for benign paroxysmal positional vertigo. This systematic review aims to comprehensively evaluate its clinical efficacy and safety.

**Methods:**

We searched eight electronic databases for randomized controlled trials (RCTs) investigating SSPR therapy for RD from database inception to 5 May 2026. The quality of methodology was evaluated by the Cochrane Risk of Bias tool (ROB 2.0). Meta-analysis was conducted using RevMan 5.4 and Stata 18.0.

**Results:**

Thirty-seven RCTs (2,794 participants) were included. Compared with standard of care (SOC) alone, SSPR therapy significantly reduced dizziness handicap inventory (DHI) scores [MD = −9.24, 95% CI: (−10.98, −7.50), *p* < 0.00001], visual analogue scale (VAS) scores [MD = −1.32, 95% CI: (−1.71, −0.93), *p* < 0.00001], and TCM syndrome scores [SMD = −1.84, 95% CI: (−2.49, −1.20), *p* < 0.00001], while achieving higher dizziness resolution rate and clinical effective rate. Furthermore, SSPR therapy plus SOC combination compared with SOC, yielded greater reductions in DHI scores [MD = −9.33, 95% CI: (−11.45, −7.21), *p* < 0.00001], VAS scores [MD = −1.39, 95% CI: (−2.20, −0.58), *p* = 0.0008], TCM syndrome scores [SMD = −1.39, 95% CI: (−1.97, −0.81), *p* < 0.00001], and recurrence rate, while elevating dizziness resolution rate and clinical effective rate. No severe adverse events were reported in the studies that reported safety outcomes.

**Conclusion:**

SSPR therapy appears effective in improving RD symptoms and may reduce recurrence rates. However, its safety remains uncertain due to incomplete reporting of adverse events across the included studies. Given the limitations of the included studies, high-quality, large-sample RCTs are still required for rigorous verification.

**Systematic Review Registration:**

http://www.crd.york.ac.uk/PROSPERO/, identifier CRD420251024692.

## 1 Introduction

As the most common vestibular cause of dizziness, benign paroxysmal positional vertigo (BPPV) is characterized by paroxysmal vertigo induced by changes in head position relative to the direction of gravity. According to a cross-sectional study (1), BPPV has a lifetime prevalence of 2.4% and a 1-year prevalence of 1.6%, predominantly affecting older populations and females. Notably, BPPV has a high recurrence rate reaching 37% after recovery, with 76% of recurrences occurring within the first two years (2). The current consensus pathogenesis of BPPV is that otolith particles detach and enter the semicircular canal lumen (canalithiasis) or adhere to the cupula (cupulolithiasis), where their gravitational displacement leads to characteristic vertigo and nystagmus (3). Canalith Repositioning Procedures (CRPs), including manual repositioning maneuvers and mechanically assisted approaches, are currently recognized as the primary and most effective treatment for BPPV (4). However, even after successful CRPs, approximately 43.0% (5, 6) of patients experience residual dizziness (RD), which manifests as nonspecific vertigo, unsteadiness, and a floating sensation, and is often complicated by insomnia and anxiety. In some cases, RD may progress to persistent postural-perceptual dizziness (PPPD) (7). RD significantly elevates fall risks, exacerbates mood disorders, diminishes patients' quality of life, and ultimately increases the socioeconomic burden. Several hypotheses have been proposed regarding the presentation of RD, involving lingering otoconial debris, concomitant vestibular disorders, incomplete central vestibular compensation, autonomic nervous dysfunction, psychological stress, vitamin D deficiency and microcirculatory dysfunction (8). In clinical practice, objective vestibular function tests, such as the video head-impulse test (vHIT) and caloric test, play a vital role in identifying underlying vestibular dysfunction, particularly when typical positional findings are no longer evident (9). In modern medicine, the treatment of RD primarily relies on pharmacological interventions such as betahistine, extract of Ginkgo biloba leaves, vitamin D supplementation, or non-pharmacological interventions like vestibular rehabilitation therapy (VRT) (4, 10). However, current therapeutic strategies for RD lack specificity, with no gold-standard treatment regime in clinical practice. The efficacy of a single treatment method has certain limitations. Therefore, further research on the mechanisms underlying RD and exploring safe, effective therapeutic strategies remain an urgent clinical priority.

In recent years, a growing number of studies have reported that spleen-strengthening and phlegm-resolving (SSPR) herbal therapy has a positive effect on RD, making it a new research hotspot (11). In traditional Chinese medicine (TCM) theory, RD is classified under the category of “vertigo”. Zhang Jingyue stated that “No dizziness arises without deficiency,” while Zhu Danxi noted that “No dizziness arises without phlegm.” This highlights that deficiency and phlegm are vital pathogenic factors contributing to RD, characterized by a pathogenesis of intermingled deficiency and excess. Clinical observations show that patients often suffer from rotational vertigo, which contributes to irregular diet. Fear about falls or concern about symptom recurrence gives rise to emotional disturbances. Additionally, elderly patients or those with chronic illness frequently present with constitutional weakness and zang-fu disfunction. The above factors lead to spleen deficiency. As the celebrated physician Li Zhongzi stated, “The spleen is the source of phlegm production.” The dysfunction of the spleen triggers the internal retention of fluid-dampness, which eventually condenses into phlegm. As a yin pathogen, phlegm-turbidity is inherently heavy, turbid and viscous, impeding the qi mechanism and disturbing the ascending-descending balance of clear yang and turbid yin. Consequently, the failure of clear yang to ascend deprives the clear orifices of nourishment, while the failure of turbid yin to descend leads to the obstruction of these orifices, resulting in dizziness and unsteady gait. Although previous systematic reviews and meta-analyses have suggested the beneficial effects of TCM in treating RD, they are generally limited by a small number of included trials. Moreover, previous studies have generally investigated TCM as a broad treatment for RD, rather than focusing on a specific therapeutic principle (12). In contrast, the present review specifically focuses on SSPR therapy, a defined TCM therapeutic approach for RD. Therefore, this study conducted a comprehensive systematic review and meta-analysis of randomized controlled trials (RCTs) on this intervention for RD. We aim to evaluate its clinical efficacy and safety, thereby providing a robust, evidence-based scientific foundation for clinical application.

## 2 Methods

### 2.1 Registration

The protocol of this review was originally registered on PROSPERO (CRD420251024692) as Version 1 on 11 August 2025, and subsequently updated as Version 2 on 14 June 2026 (Available from: http://www.crd.york.ac.uk/PROSPERO/). Between these two versions, specific amendments were made to refine the eligibility criteria, outcome definitions, and methodological procedures. Specific amendments included expanding the control group from Western medicine monotherapy to standard of care (including VRT), refining the primary outcome from “symptom disappearance rate” to “dizziness resolution rate” for better specificity, integrating the Grade of Recommendations Assessment, Development, and Evaluation (GRADE) approach to evaluate evidence certainty, and adding Stata 18.0 for publication bias and sensitivity analyses. This systematic review followed the PRISMA 2020 guideline (Supplementary Table S1).

### 2.2 Criteria for inclusion and exclusion

#### 2.2.1 Types of studies

This systematic review included RCTs evaluating the efficacy and safety of SSPR therapy for RD after repositioning of BPPV. RCTs were compared (a) SSPR therapy vs. control [placebo, standard of care (SOC), no intervention] or (b) SSPR therapy plus SOC combination vs. SOC.

#### 2.2.2 Types of participants

We included participants diagnosed with RD following successful CRPs for BPPV, according to recognized diagnostic criteria (4). Given that the success of CRPs was reported inconsistently across primary studies, successful CRP was considered as at least one of the following: (a) an explicit statement of successful repositioning by the original study authors; (b) complete resolution of positional vertigo; (c) complete resolution of positional nystagmus. Notably, most studies documented the resolution of both positional vertigo and positional nystagmus, whereas some reported only the resolution of positional vertigo or positional nystagmus. In addition, a small proportion of studies explicitly reported successful CRPs without detailing the disappearance of both positional vertigo and positional nystagmus. In all cases, primary studies confirmed that BPPV had resolved prior to RD evaluation. There are no restrictions on age, gender, race, or disease duration.

#### 2.2.3 Types of intervention

(a) Experimental group: SSPR therapy, or SSPR therapy combined with SOC. Prescriptions were required to meet either of the following criteria: (1) for classic formulas and their modifications, the prescription was officially recognized as possessing the spleen-strengthening or stomach-harmonizing properties combined with phlegm-resolving effects according to the authoritative textbook *Formulas of Traditional Chinese Medicine* (13); or (2) for self-formulated prescriptions, eligibility was determined based on herbal composition, requiring the inclusion of both recognized spleen-strengthening herbs and phlegm-resolving herbs, as defined in the authoritative textbook *Chinese Materia Medica* (14)*.* Addition and subtraction based on TCM syndrome patterns were permitted. (b) Control group: SOC, a placebo, or no intervention. SOC refers to guideline-recommended or clinically routine treatment for RD, encompassing Western medicine (WM, e.g., betahistine mesylate, flupentixol/melitracen, extract of ginkgo biloba leaves drops, and VRT). All other interventions were identical, with no other TCM.

#### 2.2.4 Types of outcomes

The dizziness handicap inventory (DHI) score, which is widely implemented in efficacy evaluations, was analyzed as a continuous variable, with lower scores indicating less self-perceived dizziness handicap and better quality of life (15, 16). Dizziness resolution rate reflects the control degree of core symptoms. It was defined as the proportion of participants with complete dizziness symptom resolution relative to the total study population.

Visual analogue scale (VAS) is also widely used in clinical research to accurately assess patients' subjective symptom intensity (17). TCM syndrome score reflects the severity of disease within the domain of syndrome patterns. The clinical effective rate reflects the aggregate therapeutic efficacy and was defined as the proportion of patients achieving clinical cure, marked effect, or effectiveness, calculated as the sum of these cases divided by the total study population, based on the criteria used in each individual trial as assessed by scale reduction rates in DHI or TCM syndrome scores. The recurrence rate was calculated using the data extracted across all reported follow-up timepoints, defined as the number of recurrent cases divided by the total study population who completed follow-up at each corresponding timepoint. For quantitative synthesis, meta-analysis of recurrence rates was strictly restricted to a standardized 6-month follow-up timepoint.

Primary outcome included the following: (a) DHI; (b) dizziness resolution rate. Secondary outcomes included the following: (a) VAS; (b) TCM syndrome score; (c) clinical effective rate; (d) recurrence rate. Safety outcomes included adverse events.

#### 2.2.5 Exclusion criteria

(a) Incomplete, inconsistent, or unavailable data or duplicate publications; (b) prescriptions lacking specified composition and dosage; (c) reporting none of the above outcomes; (d) non-human studies.

### 2.3 Search strategy

We searched eight Chinese and English databases from database inception to 5 May 2026: PubMed, Web of Science (WOS), Excerpta Medica Database (Embase), Cochrane Library, China National Knowledge Infrastructure (CNKI), Wanfang Database, Chinese Science and Technology Journals Database (VIP), and Chinese Biomedical Database (SinoMed). No limitation on language. To enhance search comprehensiveness and prevent omission due to non-standardized terminology for residual dizziness, a broad retrieval strategy combining BPPV and TCM terms was implemented. The main search terms included MeSH terms and relevant entry terms, as follows: “benign paroxysmal positional vertigo”, “benign recurrent vertigo”, “benign paroxysmal positional”, “familial vestibulopathy”, “BPPV”, “traditional Chinese medicine”, “Chinese medicine”, “traditional medicine”, “herbal therapy”, “herbal remedy”, “herbal drug”, “Chinese herbal formula”, “herbal medicine”, “Chinese herb”, “phytotherapy”, “Chinese drug”. The specific searching strategies of different databases are presented in Supplementary Table S2.

### 2.4 Study selection and data extraction

Two reviewers (YZ and BZ) independently screened the studies, extracted data, and cross-checked the results through EndNote software. Using this software, duplicate literature was removed through automated detection and manual verification. During literature screening, two reviewers first read titles and abstracts to exclude irrelevant records, and then read the full texts to determine final included studies. Subsequently, the data extraction included: (a) basic information (title, authors, journal source, publication year); (b) study design (case source, sample size, diagnostic criteria, inclusion and exclusion criteria, interventions and comparisons); (c) participant characteristics (age, sex, disease duration); (d) outcome measures. Any disagreements were resolved through consultation with a third researcher (YS).

### 2.5 Risk of bias assessment

Based on the Cochrane Risk of Bias Tool 2.0 (18), two reviewers (YZ and BZ) independently assessed the risk of bias for each outcome at the end of the intervention period in each trial across five critical domains: randomization process, deviations from intended interventions, missing outcome data, measurement of the outcome, and selection of the reported result. Each domain was graded as “low,” “some concerns,” or “high.” Disagreements between reviewers were resolved through a third reviewer (YS).

### 2.6 Data analysis

Meta-analysis of the outcomes were performed using RevMan software (version 5.4). For continuous data, the effect size was expressed as mean difference (MD) or standardized mean difference (SMD) with 95% confidence interval (CI) using the Inverse Variance (IV) method. MD was applied to outcomes using the same measurement method, while SMD was employed for outcomes using different measurement methods. For studies that only reported continuous data as medians and interquartile ranges or ranges, we converted these values into means and standard deviations using standard validated statistical formulas prior to meta-analysis (19, 20). For dichotomous data, the effect size was expressed as relative risk (RR) with 95% CI using the Mantel-Haenszel (M-H) method. Non-meta-analyzable data were presented as descriptive summaries. Heterogeneity among studies was quantified using the I-square (*I*^2^) statistic. Because of the variations in the composition, dosage, and treatment duration of TCM across the studies, we utilized a random-effects model for data synthesis, with the between-study variance (*τ*^2^) estimated using the DerSimonian-Laird method. *I*^2^ ≤ 50% indicated acceptable heterogeneity, while *I*^2^ > 50% represented substantial heterogeneity. When *I*^2^ > 50%, we performed subgroup analysis based on specific types of SOC to explore and explain the sources of heterogeneity. Sensitivity analysis was conducted to identify the specific studies contributing to heterogeneity. When the same outcome included at least 10 studies, a funnel plot and Egger's test were conducted to evaluate potential publication bias for respective outcomes. Both sensitivity analysis and publication bias evaluation were performed by Stata 18.0. The quality of evidence was assessed using the GRADE approach (21) and categorized as high, moderate, low, or very low for each outcome.

## 3 Results

### 3.1 Search result

A total of 2,437 articles were retrieved through the initial search, of which 1,171 were duplicated. After reviewing the titles and abstracts, 108 articles were retained based on inclusion and exclusion criteria such as study design, intervention, and participants. Following a full-text review, 71 studies were excluded for the following reasons: 37 studies were not SSPR intervention, 3 studies were not RCTs, 3 studies lacked specified dosage, 1 study lacked availability of outcome data, 23 studies failed to meet the diagnostic criteria, 2 studies was duplicate, 1 study had inconsistent data and 1 study was not on RD patients. Finally, 37 studies were included. The selection process is shown in Figure 1.

**Figure 1:**
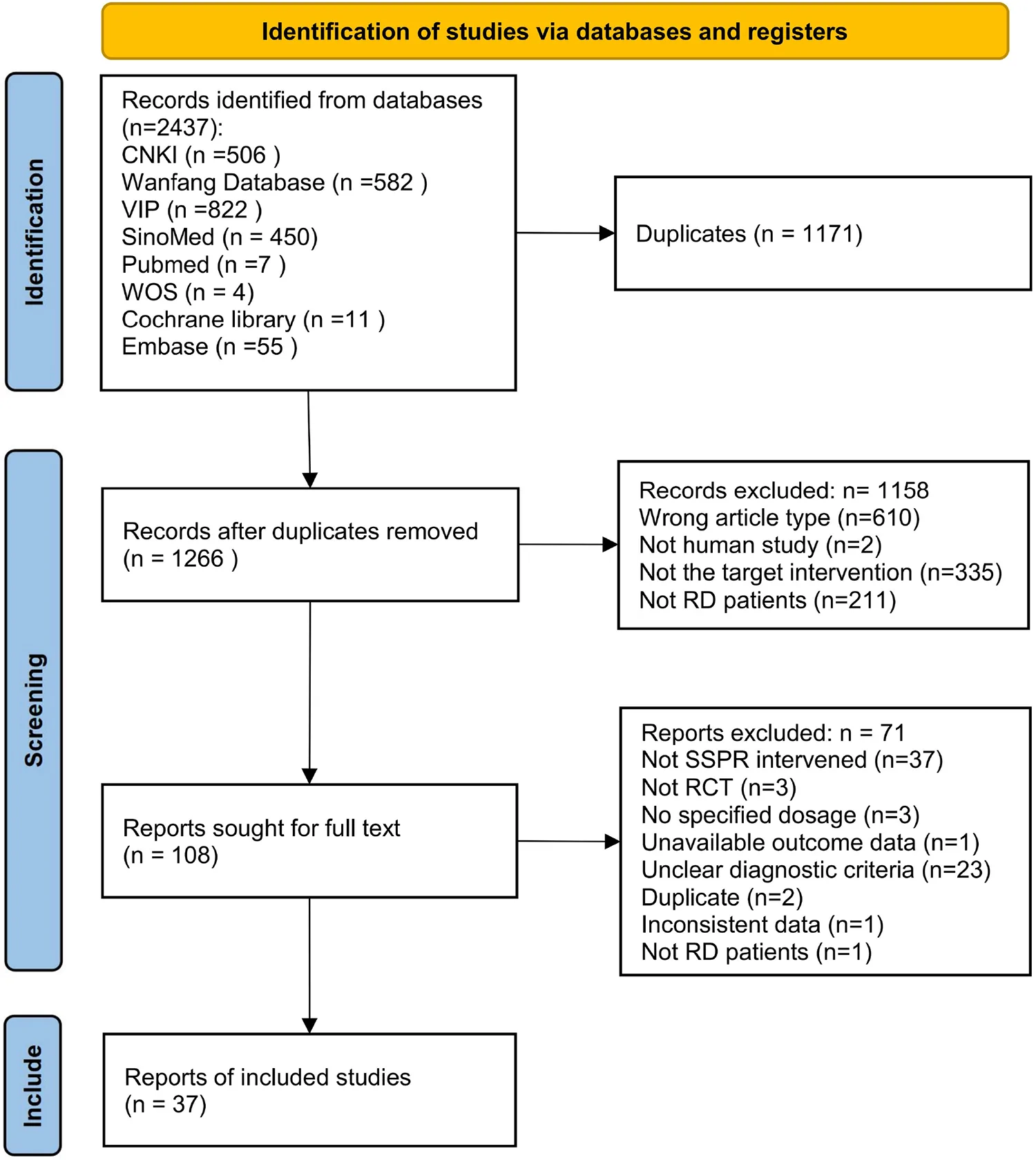
PRISMA flowchart of literature selection process.

### 3.2 Study characteristics

Thirty-seven RCTs (10, 11, 22–56), were included, all of which were published in Chinese between 2016 and 2026. The details of the included trials are summarized in Table 1. The frequency and classification of Chinese herbs are shown in Table 2, and the specific ingredients and dosages of SSPR prescriptions are detailed in Supplementary Table S3. A total of 2,794 participants were involved, with 1,337 in the experimental group and 1,457 in the control group. Notably, 3 trials utilized a three-arm design (26, 37, 38), and duplicate counts within the experimental groups were excluded to avoid double-counting. The sample size of individual trials ranged from 42 to 200 cases. The male-to-female ratio of the included participants was 1,136:1,661. One study (55) reported gender distribution only for the baseline randomized population without specifying the gender of the three dropouts for the final analyzed population. Therefore, baseline data were recorded from this trial, which was deemed unlikely to introduce substantial bias in the description of the overall baseline gender characteristics given the minimal dropout rate (3/80, 3.75%). Because of the three three-arm trials involving multiple comparators, a total of 40 entries were generated across the 37 trials, involving 4 distinct comparison groups: SSPR therapy vs. no intervention, SSPR therapy vs. placebo, SSPR therapy vs. SOC, and SSPR therapy plus SOC combination vs. SOC. Among them, two three-arm trials (26, 38) were categorized into different comparison groups based on control types and analyzed separately, so their samples were not double-counted. For the trial (37) within the same comparison group, the sample size of the shared arm was split equally to prevent weight bias.

**Table 1:** Characteristics of the included trials.

Study ID	Sample size (T/C)	Sex (M/F)	Age/year	Intervention		Treatment duration/d	Outcomes
				T	C		
SSPR therapy vs. no intervention							
Fan SH 2020	30/30	T:9/21 C:11/19	T:56.27 ± 11.95 C:58.63 ± 12.30	Pingxuan Dingyun Decoction	–	14	①②④⑤⑦⑥
Liu J 2022	100/100	T:26/74 C:26/74	T:54 ± 13 C:53 ± 11	TCM	–	14	①②④⑤⑦
Shu F 2022	30/31	T:10/20 C:11/20	T:56.93 ± 10.76 C:54.97 ± 13.38	Xuandekang Granule	–	28	①③⑦
Zhang Y *et al.* 2021	45/45	T:22/23 C:20/25	T:59.34 ± 10.4 C:57.15 ± 12.57	Huatan Tongnao Decoction	–	14	①②
SSPR therapy vs. placebo							
Liu S *et al.* 2025a	28/28	T:11/17 C:12/16	T:47.36 ± 4.87 C:50.89 ± 5.41	Tianma Jiannao Granule + VRT	Placebo + VRT	28	①②③④⑤
Wu WL 2023	36/38	T:13/23 C:15/23	T:47.72 ± 13.71 C:50.61 ± 14.51	Tongqiao Jingxuan Decoction	placebo	14	①④⑤⑦
Zhu YJ 2021	29/26	T:12/17 C:8/18	T:51.90 ± 13.29 C:58.69 ± 14.31	Jianpi Huayin Decoction	placebo	14	①②④⑤⑦
SSPR therapy vs. SOC							
Cai HC *et al*2016	30/30	T:15/15 C:13/17	T:49.23 ± 12.04 49.65 ± 12.42	Modified Xiaqi Decoction	Flupentixol/Melitracen + Conventional WM	10	①④
Huang SH 2020	38/38	T:17/21 C:16/22	T:45.50 ± 10.00 C:48.74 ± 9.26	Zhiyun Huatan Decoction	BHT-M	28	①②④⑤⑦
Huang ST *et al*2021	39/39	T:16/23 C:17/22	T:48.0 ± 8.6 C:50.0 ± 9.1	Modified BBTD	Extract Of Ginkgo Biloba Leaves Drops	14	①②③
Huang Y *et al*2017	30/30	T:12/18 C:16/14	T:39.96 ± 3.42 C:40.66 ± 2.95	Modified Wendan Decoction	BHT-M	14	①⑦
Lin XQ 2019	30/30	T:11/19 C:8/22	T:50.30 ± 9.083 C:50.13 ± 9.997	Modified BBTD	BHT-M	14	①③④⑤⑦
Liu YF *et al*2026	30/30	T:9/21 C:7/23	T:53.77 ± 9.72 C:53.83 ± 10.15	Qingqiao Dingxuan Decoction	BHT-M	14	①③④⑤
Qin ZG 2020	30/30	28/32	50.8	Modified BBTD combined with Taohong Siwu Decoction	BHT-M	14	②⑦
Shao DF *et al*2023	21/21	T:11/10 C:13/8	T:46.23 ± 1.23 C:47.13 ± 0.24	Wenzhong Dingxuan Decoction	Paroxetine + BHT-M	28	①
Wang DD *et al*2019a	40/40	67/53	48.3 ± 12.4	Dingxuan Granule	BHT-M	14	④⑦
Wang DD *et al*2019b	40/40			Dingxuan Granule	VRT	14	④⑦
Wang HG *et al*2016a	60/60	T:22/38 C:21/39	T:62.3 C:61.6	Xiaqi Decoction + Conventional WM	Flupentixol/Melitracen + Conventional WM	14	①④⑦
Wang XH 2024	30/30	T:11/19 C:9/21	T:67.63 ± 5.49 C:68.80 ± 5.94	Guizhi Plus Gegen Decoction combined with Danggui Shaoyao Powder + Baseline treatment	Betahistine Hydrochloride + Baseline treatment	14	①②③④⑤⑦
Wang ZE 2022	45/44	T:19/26 C:16/28	T:55.13 ± 7.70 C:54.32 ± 8.89	Dingxuan Decoction	BHT-M	14	①③④⑤⑦
Zhang DQ 2020	39/38	T:22/17 C:22/16	T:53.48 ± 16.42 C:52.45 ± 15.36	Modified BBTD	Escitalopram Oxalate	14	①⑤
Zhang JW 2019	30/30	T:10/20 C:13/17	T:66.70 ± 8.00 C:65.77 ± 8.88	Added BBTD	BHT-M	14	①③④⑤⑦
Zhou JJ 2022	34/31	T:14/20 C:9/20	T:54.82 ± 13.25 C:54.10 ± 12.47	Dingxuan Decoction	BHT-M	14	①③④⑤⑦
SSPR therapy plus SOC combination vs. SOC							
Cheng JH 2022	30/30	T:11/19 C:9/21	T:52.17 ± 13.20 C:51.30 ± 13.24	Added Yuzhu Dingxuan Decoction + BHT-M + Baseline treatment	BHT-M + Baseline treatment	14	①②④⑤⑦
Ding WJ *et al*2019	32/28	T:15/17 C:12/16	T:57.43 ± 12.17 C:57.18 ± 13.45	BBTD + Conventional WM	Conventional WM	5	①
Gong XH 2024	34/32	T:14/20 C:11/21	T:51.50 ± 9.97 C:53.16 ± 10.23	Added Jiawei Linggui Zhugan Decoction + BHT-M	BHT-M	14	①④⑤⑥⑦
Guo MZ *et al*2025	39/39	T:18/21 C:20/19	T:67.55 ± 5.69 C:67.88 ± 5.33	Modified Dingxuan Zhiyun Decoction + BHT-M + VRT	BHT-M + VRT	14	①⑥⑦
Li XQ *et al*2018	52/53	T:23/29 C:21/32	T:53.04 ± 9.51 C:54.41 ± 8.65	Xuanyun Formula + BHT-M	BHT-M	7	①②⑥⑦
Liu S *et al*2025b	28/28	T:11/17 C:14/14	T:47.36 ± 4.87 C:48.64 ± 4.21	Tianma Jiannao Granule + VRT	VRT	28	①②③④⑤
Qiu YJ *et al*2024	36/36	T:13/23 C:14/22	T:43.15 ± 12.26 C:41.28 ± 11.35	Modified Wendan Decoction + BHT-M	BHT-M	14	①④⑤⑥⑦
Tong XP 2024	31/32	T:9/22 C:11/21	T:51．74 ± 8．61 C:50．75 ± 8．78	Jieyu Huatan Decoction + BHT-M + Flupentixol/Melitracen	BHT-M + Flupentixol/Melitracen	14	①④⑤⑦
Wang B *et al*2022	30/30	T:14/16 C:13/17	T:51.32 ± 5.82 C:51.26 ± 5.74	BBTD + BHT-M	BHT-M	14	①④⑤
Wang HG *et al*2016b	60/60	T:22/38 C:20/40	T:62.3 C:63.2 T:66.59 ± 3.45 C:66.61 ± 3.48	Xiaqi Decoction + Conventional WM	Conventional WM	14	①④⑦
Wang R *et al*2024	41/41	T:24/17 C:26/15		Added BBTD + BHT-M	BHT-M	14	①②⑤⑦
Xu Z *et al*2024	24/24	T:8/16 C:10/14	T:65.96 ± 6.03 C:64.83 ± 7.97	Buzhong Yiqi Decoction combined with Erchen Decoction + Betahistine	Betahistine	7	④⑤
Yu M 2024	30/30	T:13/17 C:9/21	T:51.07 ± 15.00 C:57.20 ± 14.45	Added Wendan Decoction + Conventional WM	Conventional WM	28	①②③④⑤⑥⑦
Zeng YS *et al*2024	30/30	T:13/17 C:14/16	T:47.35 ± 6.57 C:48.23 ± 8.68	Modified BBTD + VRT	VRT	28	①④
Zhang XX 2024	36/36	T:15/21 C:16/20	T:51.3 ± 7.12 C:52.19 ± 6.81	Added BBTD + BHT-M	BHT-M	14	①④
Zhang Y *et al*2023	38/39	T:13/27* C:14/26*	T:65.0 ± 8.2 C:66.0 ± 7.8	Xuanfu Daizhe Decoction + BHT-M	BHT-M	14	①②⑥
Zhou YJ *et al*2025	30/30	T:13/17 C:16/14	T:57.78 ± 4.49 C:58.36 ± 4.85	Huatan Pingxuan Formula + BHT-M	BHT-M	7	①③⑤

BBTD, Banxia Baizhu Tianma Decoction; BHT-M, Betahistine Mesylate; C: control group; d, day; F, female; M, male; SOC, Standard of care; SSPR, Spleen-strengthening and phlegm-resolving; T: experimental group; VRT, Vestibular rehabilitation therapy; WM, Western medicine. ① DHI; ② Dizziness resolution rate; ③ VAS; ④ TCM syndrome score;⑤ Clinical effective rate; ⑥ Recurrence rate; ⑦ Adverse events.

* Gender distribution was extracted from the baseline randomized population, as the gender distribution of the final analyzed population was not separately reported.

**Table 2:** Frequency and classification of Chinese herbs.

Chinese herbs	Nature	Frequency	Classification	Rate %
Pinelliae Rhizoma (Banxia)	Warm	34	Dampness-eliminating and phlegm-resolving herbs	8.81
Poria (Fuling)	Neutral	30		7.77
Rhizoma Alismatis (Zexie)	Cold	21		5.44
Atractylodis Macrocephalae Rhizoma (Baizhu)	Warm	31	Spleen-strengthening and qi-tonifying herbs	8.03
Glycyrrhizae Radix et Rhizoma (Gancao)	Neutral	31		8.03
Jujubae Fructus (Dazao)	Warm	15		3.89
Gastrodiae Rhizoma (Tianma)	Neutral	28	Liver-calming and wind-dispelling herbs	7.25
Citri Reticulatae Pericarpium (Chenpi)	Warm	20	Qi-regulating herbs	5.18
Citri Grandis Exocarpium (Juhong)	Warm	10		2.59
Zingiberis Rhizoma Recens (Shengjiang)	Slightly warm	13	Exterior-releasing herbs	3.37
Acori Tatarinowii Rhizoma (Shichangpu)	Warm	12	Orifice-opening herbs	3.11
Chuanxiong Rhizoma (Chuanxiong)	Warm	10	Blood activiating and stasis dissolving	2.59

Herbs with a usage frequency at least 10 times are presented in this table.

### 3.3 Risk of bias

Risk of bias was evaluated independently for each outcome across all 37 included trials. The risk of bias summary for the two primary outcomes is presented in Figure 2. Regarding the randomization process, 25 trials used the random number table, while 12 trials did not report the randomization method. Only 7 trials performed allocation concealment, of which two mentioned blinding. Thirteen studies reported case dropouts. Among them, only two trials had dropout rates under 5% with dropouts unrelated to intervention allocation, and others including 2 double-blind trials had deviations from the intended interventions and were rated as “high” risk of bias. Regarding the missing outcome data, 24 trials had no dropouts, 4 trials had dropout rates below 5%, and 3 trials were stated by authors that the dropouts were missing completely at random. These 31 studies were rated as “low” risk of bias, while the remaining 6 studies were rated as “high” risk of bias due to the missing data that were outcome-related or of unknown reasons. Regarding the outcome measurement, risk of bias varied depending on the outcome indicators. For scale-based outcomes, including DHI, VAS, and TCM syndrome scores, as well as clinical effective rate, which was defined based on scale reduction rates, non-blinded trials were rated as having “some concerns”. In contrast, for dizziness resolution rate and recurrence rate, only trials that relied solely on patients' subjective descriptions were rated as “high” risk of bias. Regarding the selection of the reported result, the majority of trials were rated as “low” risk of bias, as their reported results were consistent with the pre-specified plans. Exceptions included one trial (23) which was assessed as having “some concerns” for omitting some pre-specified outcomes, and another trial (46) which was rated as “high” risk of bias owing to the selective calculation of recurrence rate using only effective patients. Further details are shown in Supplementary Table S4.

**Figure 2:**
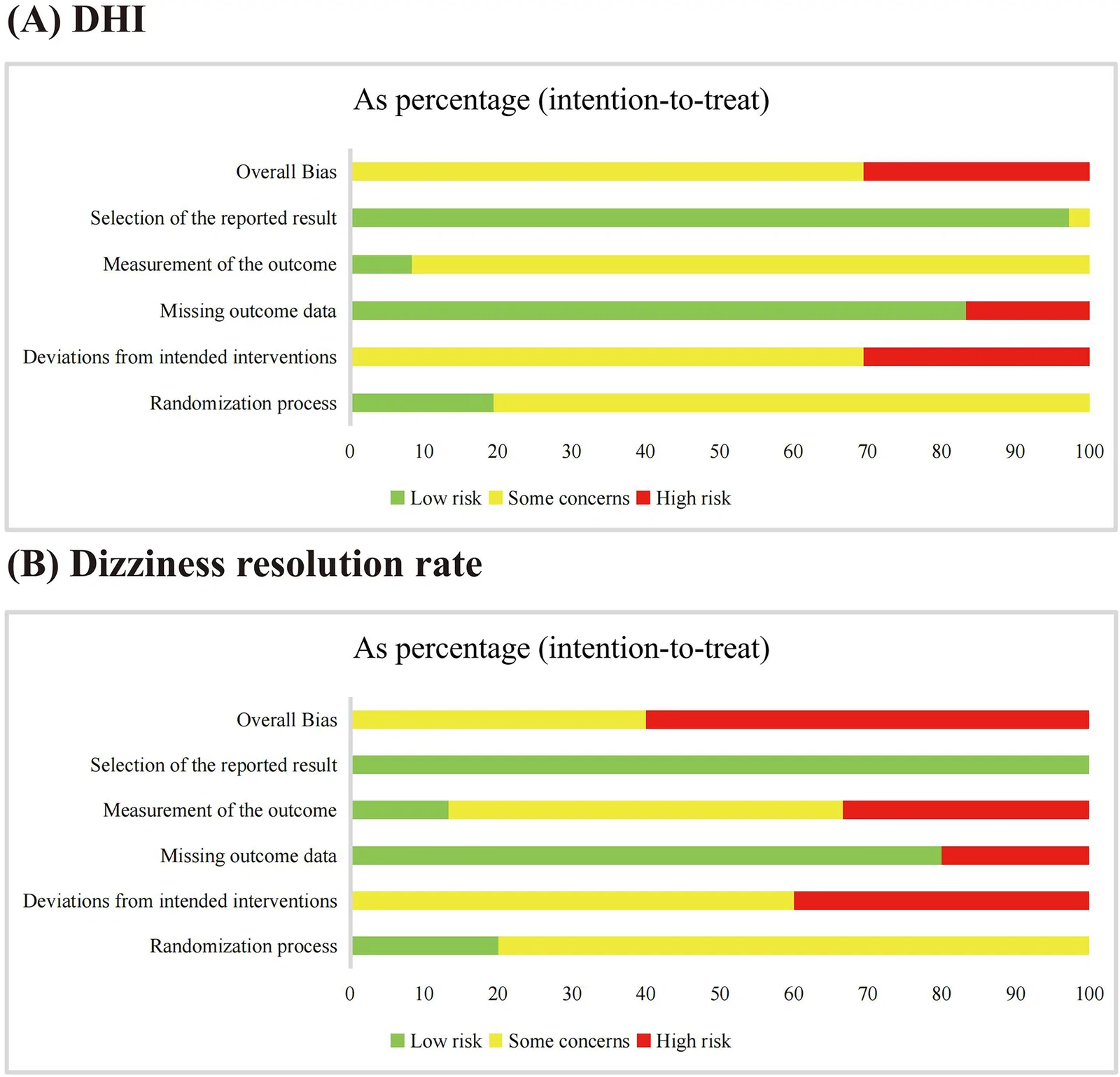
Risk of bias summary for DHI and dizziness resolution rate.

### 3.4 Meta-analysis results

#### 3.4.1 Outcomes of SSPR therapy vs. no intervention

SSPR therapy showed a significantly greater reduction compared to no intervention in DHI score [MD = −6.95, 95% CI: (−13.52, −0.37), *N* = 4, *p* = 0.04]. Additionally, data from two separate individual studies (22, 24) demonstrated that SSPR therapy significantly reduced VAS score [MD = −1.00, 95% CI: (−1.78, −0.22), *p* = 0.01] and recurrence rate [RR = 0.13, 95% CI: (0.02, 0.94), *p* = 0.04]. However, no statistically significant differences were observed regarding the dizziness resolution rate, TCM syndrome score, and clinical effective rate, which may be attributed to the limited number of included trials.

#### 3.4.2 Outcomes of SSPR therapy vs. placebo

SSPR therapy was superior to placebo in reducing DHI score [MD = −10.57, 95% CI: (−14.89, −6.25), *N* = 3, *p* < 0.00001], TCM syndrome score [SMD = −1.19, 95% CI: (−1.74, −0.64), *N* = 3, *p* < 0.0001], and improving clinical effective rate [RR = 1.40, 95% CI: (1.11, 1.78), *N* = 3, *p* = 0.005]. Additionally, data from a single study (26) showed that SSPR therapy decreased the VAS score [MD = −0.93, 95% CI: (−1.76, −0.10), *p* = 0.03]. Concurrently, SSPR therapy showed no significant differences compared with placebo in dizziness resolution rate.

#### 3.4.3 Outcomes of SSPR therapy vs. SOC

All outcomes showed statistically significant differences in comparison of SSPR therapy vs. SOC. SSPR therapy achieved lower DHI score [MD = −9.24, 95% CI: (−10.98, −7.50), *N* = 13, *p* < 0.00001], VAS score [MD = −1.32, 95% CI: (−1.71, −0.93), *N* = 7, *p* < 0.00001] and TCM syndrome score [**SMD = −1.84,** 95% CI**: [−2.49, −1.20],** *N* = 11, *p* < 0.00001; sample size of the shared experimental group in a three-arm trial (37) was split equally], alongside higher dizziness resolution rate [RR = 1.56, 95% CI: (1.15, 2.11), *N* = 4, *p* = 0.004] (Figure 3A) and clinical effective rate [**RR** **=** **1.19,** 95% CI**: (1.12, 1.28), *N*** **=** **8,** *p* < 0.00001].

**Figure 3:**
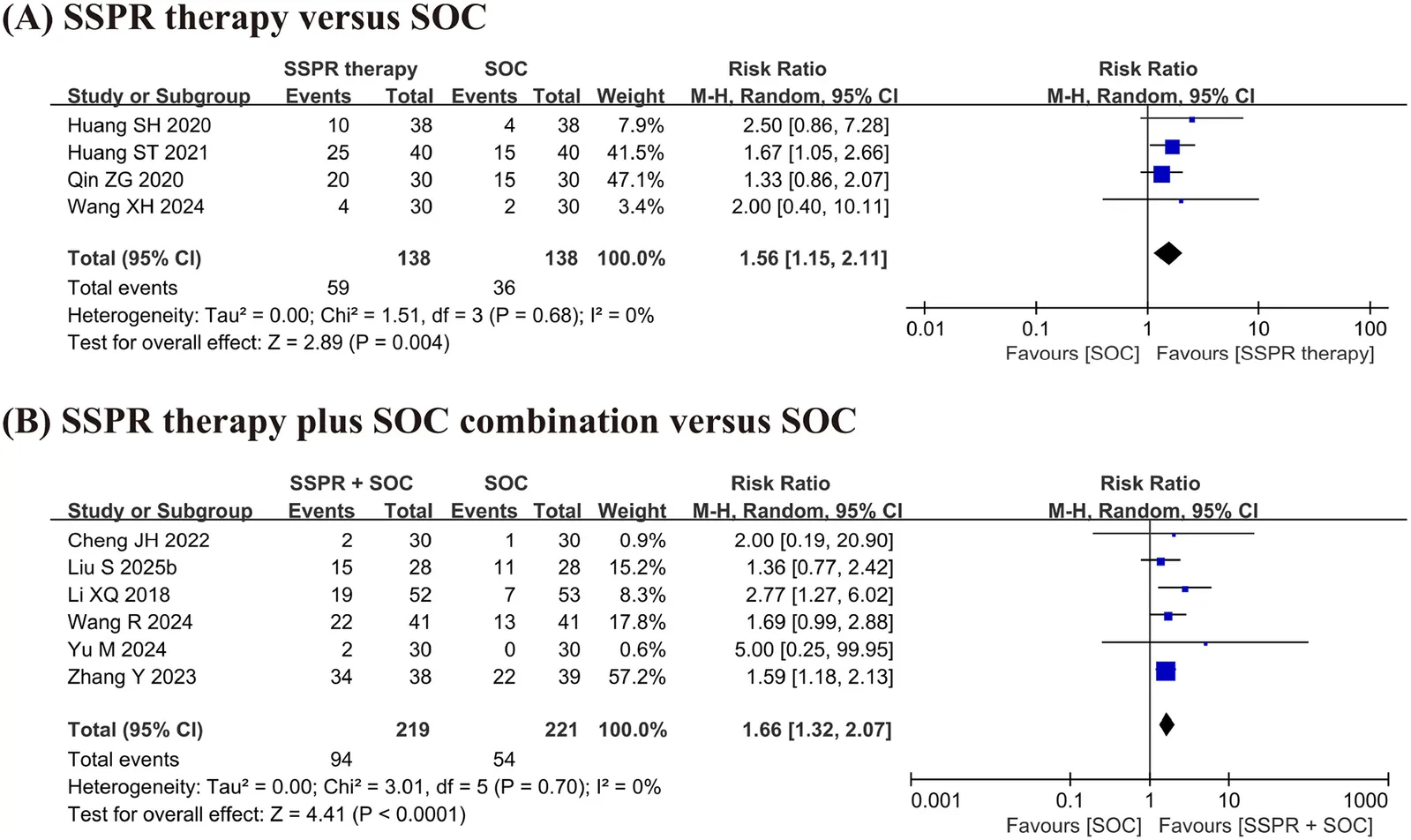
Forest plot for the meta-analysis of dizziness resolution rate.

#### 3.4.4 Outcomes of SSPR therapy plus SOC combination vs. SOC

Compared with SOC alone, SSPR therapy plus SOC combination was associated with statistically significant improvements across all evaluated outcomes. SSPR therapy plus SOC combination provided a greater reduction in DHI score [MD = −9.33, 95% CI: (−11.45, −7.21), *N* = 16, *p* < 0.00001], VAS score [MD = −1.39, 95% CI: (−2.20, −0.58), *N* = 3, *p* *=* 0.0008] and TCM syndrome scores [SMD = −1.39, 95% CI: (−1.97, −0.81), *N* = 11, *p* < 0.00001]; concurrently, it demonstrated a significantly higher dizziness resolution rate [RR = 1.66, 95% CI: (1.32, 2.07), *N* = 6, *p* < 0.0001] (Figure 3B) and clinical effective rate [RR = 1.18, 95% CI: (1.11, 1.25), *N* = 10, *p* < 0.00001] (Figure 4).

**Figure 4:**
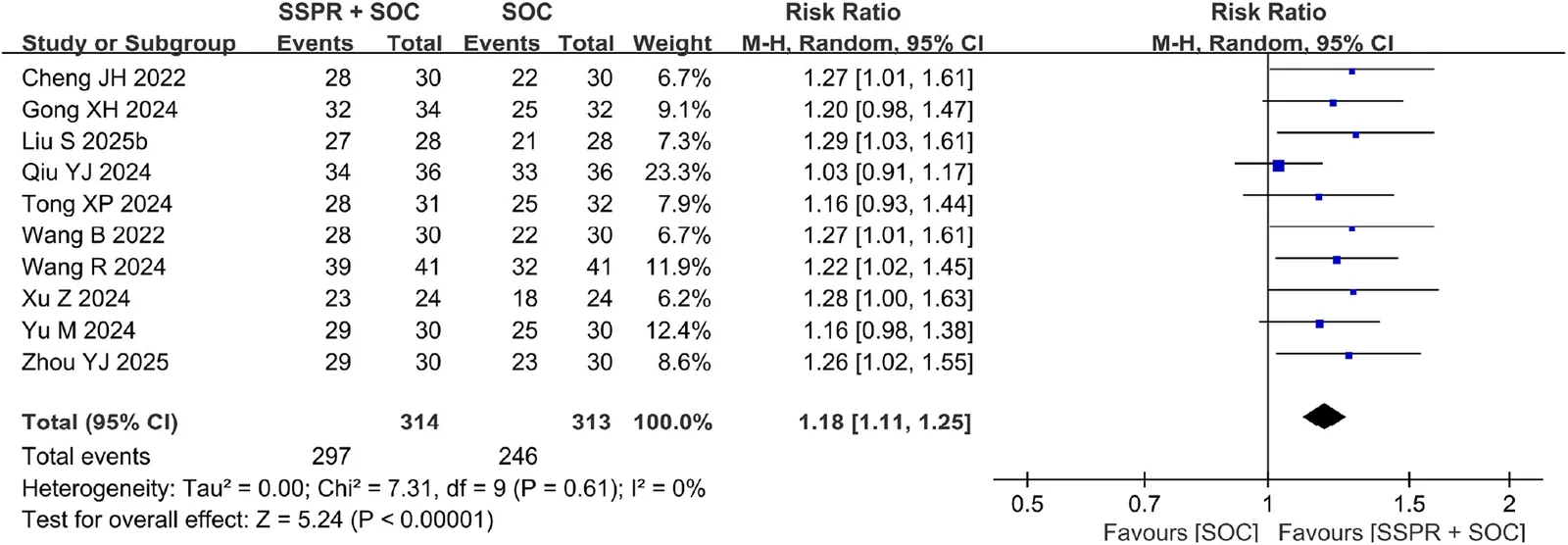
Forest plot for the meta-analysis of clinical effective rate in comparison of SSPR therapy plus SOC combination vs. SOC.

At the 6-month follow-up, pooled results showed that the SSPR therapy plus SOC combination group achieved a lower recurrence rate compared with the SOC group [RR = 0.35, 95% CI: (0.15, 0.78), *N* = 4, *p* *=* 0.01]. The recurrence rate was 4.24% in the SSPR therapy plus SOC combination group and 14.97% in the SOC group. The detailed event counts and recurrence rates for all follow-up durations are listed in Supplementary Table S5. The detailed findings of all meta-analyses are visually presented in Supplementary Table S6.

### 3.5 Subgroup analysis

Subgroup analyses were performed based on three factors.

Firstly, subgroup analyses were performed based on specific types of SOC. In SSPR therapy vs. SOC comparison, results of all subgroup analyses were consistent with the overall results. For DHI score, SSPR therapy significantly outperformed the vestibular monotherapy [MD = −8.83, 95% CI: (−10.84, −6.82), *N* = 8, *p* < 0.00001] and central psychotropic-containing regimen [MD = −11.35, 95% CI: (−14.05, −8.64), *N* = 4, *p* < 0.00001]. A single-study (31) also favored SSPR therapy over standardized phytopharmaceutical monotherapy [MD = −5.56, 95% CI: (−7.71, −3.41), *p* < 0.00001] (Figure 5A). For TCM syndrome score, SSPR therapy was superior to vestibular monotherapy [SMD = −1.37, 95% CI: (−1.98, −0.77), *N* = 8, *p* < 0.00001] and central psychotropic-containing regimen [SMD = −2.62, 95% CI: (−3.84, −1.39), *N* = 2, *p* < 0.0001]. Similarly, a single-study estimate (37) indicated superior efficacy of SSPR therapy over VRT [SMD = −4.03, 95% CI: (−4.95, −3.11), *p* < 0.00001]. Similarly, in SSPR therapy plus SOC combination vs. SOC comparison, subgroup analyses yielded results that were consistent with the overall results. SSPR therapy plus SOC combination significantly reduced DHI scores compared with WM [MD = −8.77, 95% CI: (−10.86, −6.68), *N* = 13, *p* < 0.00001] and VRT [MD = −7.88, 95% CI: (−9.52, −6.25), *N* = 2, *p* < 0.00001]. In addition, a single-study estimate (47) demonstrated its advantage over WM plus VRT combination [MD = −20.10, 95% CI: (−22.12, −18.08), *p* < 0.00001] (Figure 5B). For TCM syndrome score, SSPR therapy plus SOC combination achieved greater improvements in TCM syndrome scores across both SOC types, including WM [SMD = −1.50, 95% CI: (−2.21, −0.80), *N* = 9, *p* < 0.0001] and VRT [SMD = −0.90, 95% CI: (−1.48, −0.33), *N* = 2, *p* = 0.002].

**Figure 5:**
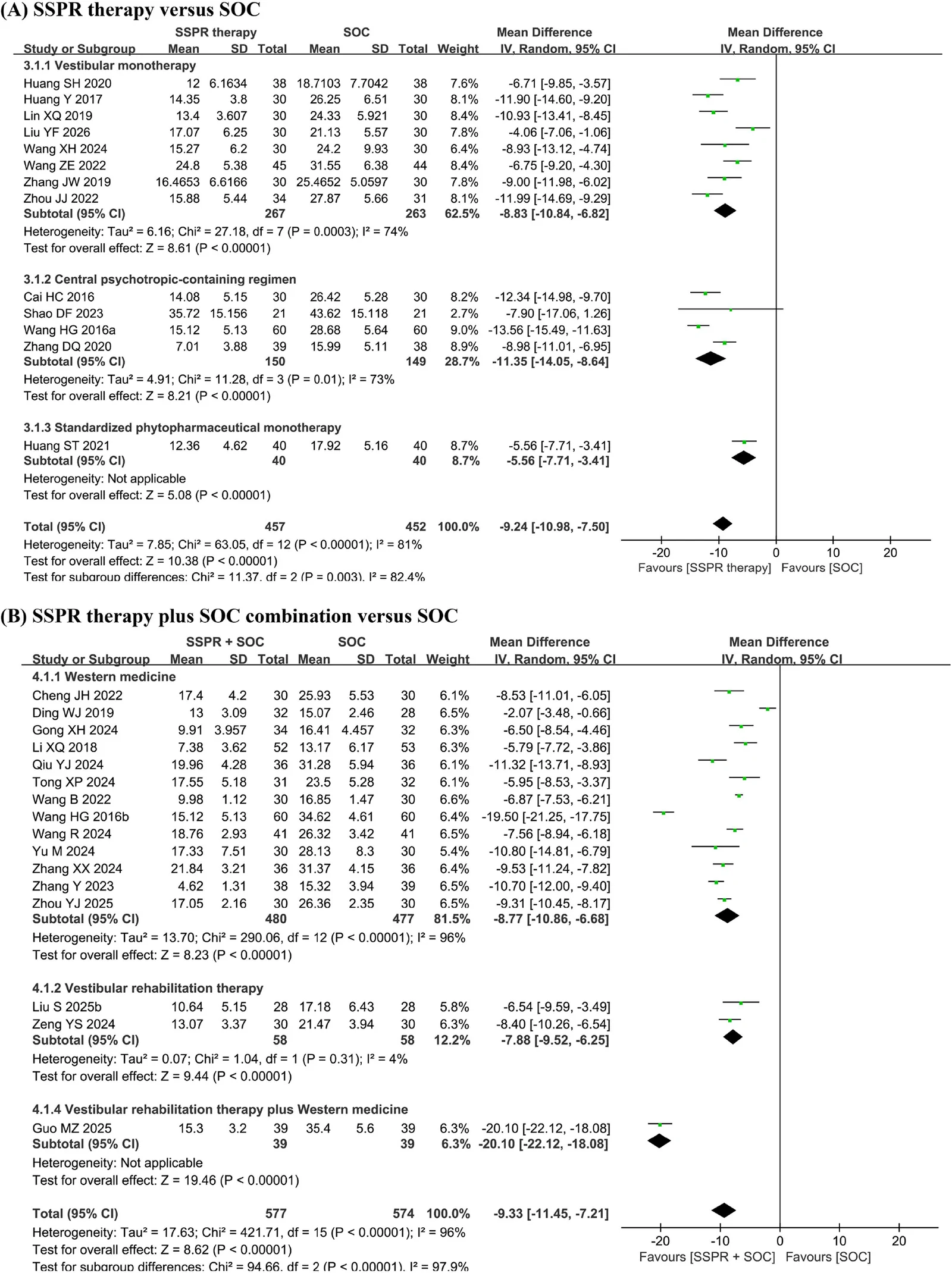
Forest plot of subgroup analyses of DHI according to specific types of SOC. Categories containing only one study represent single-study estimates rather than pooled subgroup evidence.

Secondly, subgroup analyses based on the number of herbal components significantly favored the SSPR group and the SSPR plus SOC combination group over SOC. In SSPR therapy vs. SOC comparison, SSPR therapy demonstrated statistically significant superiority over SOC in DHI (Supplementary Figure 1.A) and TCM syndrome scores (all *p* < 0.05). Similarly, in SSPR therapy plus SOC combination vs. SOC comparison, SSPR therapy plus SOC was significantly superior to SOC in both DHI [number < 10: MD = −10.04, 95% CI: [−13.54, −6.54], *N* = 9; number ≥ 10: MD = −8.41, 95% CI: [−9.49, −7.33], *N* = 7; both *p* < 0.05, Supplementary Figure S1.B] and TCM syndrome scores [number <10: SMD = −2.41, 95% CI: [−3.86, −0.96] *N* = 4; number ≥ 10: SMD = −0.83, 95% CI: [−1.03, −0.63], *N* = 7; both *p* < 0.05]. Notably, heterogeneity was markedly reduced in the subgroup with ≥ 10 herbal components. Subgroup interactions were non-significant across most comparisons (*P*_interaction_ > 0.05), except for TCM syndrome scores in SSPR plus SOC combination comparison, which exhibited moderate subgroup heterogeneity (*P*_interaction_ = 0.03).

Thirdly, subgroup analyses based on the treatment duration yielded results robustly consistent with the primary analyses. In reducing both DHI and TCM syndrome scores, SSPR group and SSPR plus SOC combination group were superior to SOC group (all *p* < 0.05), with no statistically significant subgroup interactions detected (all *P*_interaction_ > 0.05). Subgroup analysis of TCM syndrome scores for SSPR vs. SOC group was not conducted due to limited study availability.

Subgroup analysis revealed reduced heterogeneity within some subgroups, suggesting that the SOC type, treatment duration, and the number of herbal components may be sources of heterogeneity; however, *I*^2^ > 50% was still observed in some subgroups, which may be related to the diversity of SSPR prescriptions. All subgroup analyses are detailed in Supplementary Table S7.

### 3.6 Sensitivity analysis

A sensitivity analysis was performed on two primary outcomes (DHI score and dizziness resolution rate) as well as TCM syndrome score, which showed high heterogeneity. To ensure statistical reliability, these analyses were confined to comparison groups that contained five or more included studies. The results demonstrated that both outcomes remained relatively robust in comparisons of both SSPR therapy and SSPR therapy plus SOC combination against SOC (Figure 6, Supplementary Figures 2, 3).

**Figure 6:**
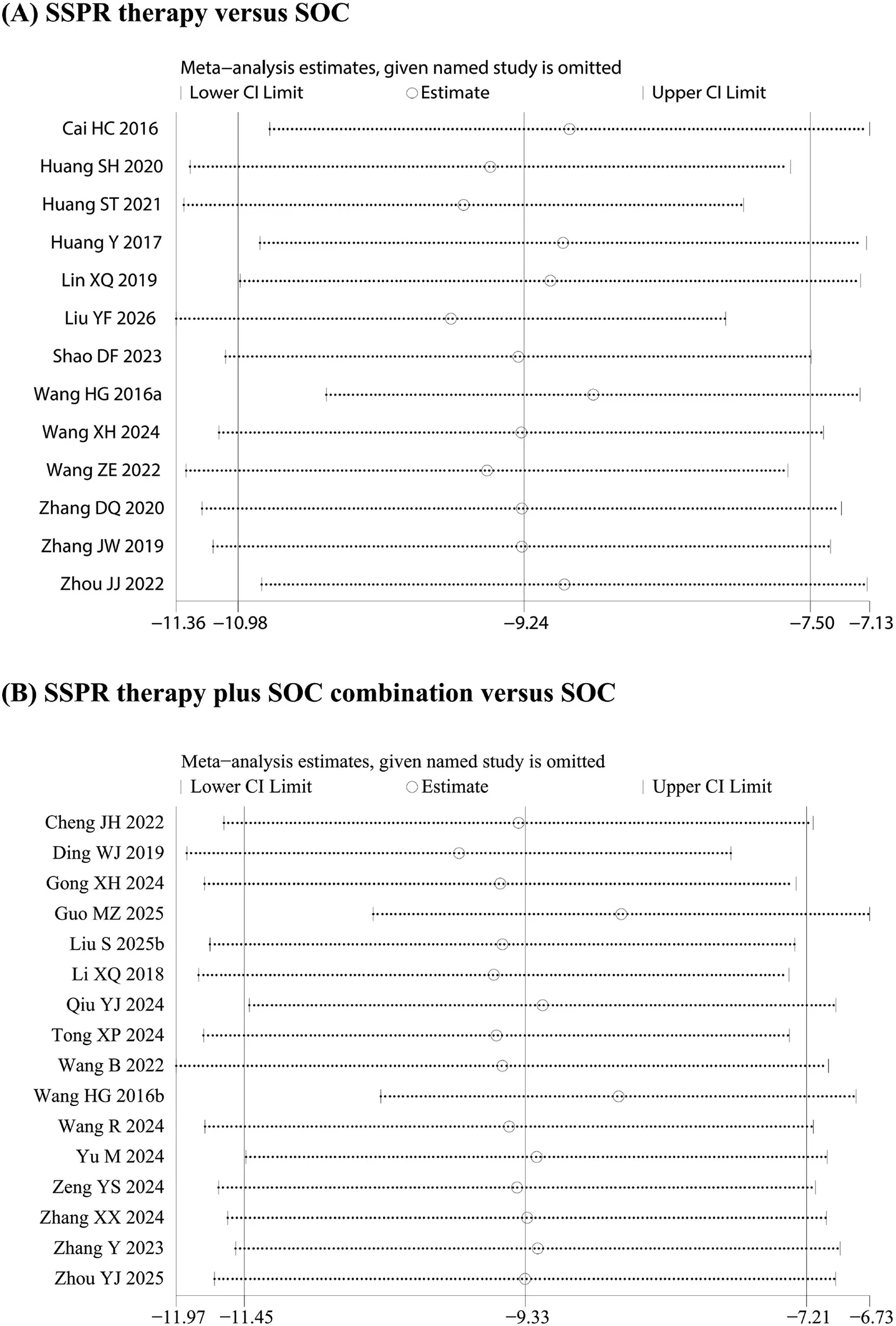
Sensitivity analysis of DHI.

Furthermore, a sensitivity analysis was carried out for the recurrence rate due to the unclear definition of the recurrence indicator in one study (47). After excluding this study, no statistically significant difference was observed between the experimental and control groups [RR = 0.40, 95% CI: (0.15, 1.07), *N* = 3, *p* *=* 0.07]; however, the lower recurrence rate persisted in the experimental group (3.97%) compared with the control group (13.28%). This loss of statistical significance may be primarily attributed to the reduction in sample size and event numbers.

### 3.7 Publication bias

Regarding the DHI scores, visual inspection of the funnel plots revealed a relatively symmetrical distribution of scatter points for both SSPR therapy vs. SOC group and SSPR therapy plus SOC combination vs. SOC group. Furthermore, Egger's test indicated no statistically significant difference for both comparisons: SSPR therapy vs. SOC group (*p* = 0.542) and SSPR therapy plus SOC combination vs. SOC group (*p* = 0.305), suggesting a low risk of publication bias in this outcome.

By contrast, funnel plots exhibited asymmetrical distributions of scatter points for several secondary outcomes. Significant asymmetry was observed for TCM syndrome score and clinical effective rate in both SSPR therapy vs. SOC comparison and SSPR therapy plus SOC combination vs. SOC comparison (both *p* < 0.05; Supplementary Figures 4, 5). A potential explanation for these findings is the variation in TCM syndrome rating scales and the subjectivity of evaluation criteria across studies.

### 3.8 Certainty of evidence

The GRADE system was utilized to assess the quality of evidence for all outcomes. Due to risks of bias, including unclear concealment and lack of blinding and incomplete outcome data, the quality of evidence for all outcomes was degraded by 1 to 2 levels. In SSPR therapy vs. no-intervention group, DHI, VAS and recurrence rate were rated as low quality, while others were rated as very low quality. In SSPR therapy vs. placebo group, all outcomes were degraded due to the limited sample size, with a low quality of evidence for VAS and very low quality for all other outcomes. In SSPR therapy vs. SOC group, VAS and clinical effective rate were rated as moderate quality, TCM syndrome score was rated as very low quality, the remaining outcomes were rated as low quality. In SSPR therapy plus SOC combination vs. SOC group, DHI was rated as moderate quality, clinical effective rate, and TCM syndrome score were rated as low quality, while the others were rated as very low quality. Supplementary Table S8 presents a comprehensive GRADE evidence assessment detailing the quality of evidence for each outcome.

### 3.9 Adverse events

Twenty-four studies mentioned adverse events. Among them, 14 studies reported no adverse events in either group, while the remaining 10 reported adverse events. Notably, one study (55) only descriptively reported dropouts due to adverse events without providing a complete adverse event profile for both groups. Therefore, it was excluded from the quantitative safety meta-analysis and only summarized descriptively. Specifically, this study reported that 1 patient in the control group withdrew from the trial due to gastric discomfort after medication administration, with no other safety data provided. SSPR therapy exhibited a lower incidence of adverse events than both no-intervention [RR = 0.52, 95% CI: (0.05, 5.40), *p* = 0.58] and SOC [RR = 0.26, 95% CI: (0.07, 1.06), *p* = 0.06]. Meanwhile, SSPR therapy plus SOC combination also presented a slightly higher incidence of adverse events compared with SOC [RR = 1.22, 95% CI: (0.48, 3.09), *p* = 0.68]. No adverse events occurred in SSPR therapy vs. placebo comparison. Overall, no statistically significant difference was observed between experimental group and control group. Adverse events in both groups were predominantly categorized as gastrointestinal symptoms, such as nausea, abdominal distension, and diarrhea, with no severe adverse events documented. Laboratory abnormalities were reported in one trial (47), which documented abnormal liver and renal function. The details were presented in Supplementary Table S9.

## 4 Discussion

### 4.1 Summary of evidence

A total of 37 trials involving 2,794 participants were included in this study to evaluate the efficacy and safety of SSPR therapy for RD. Overall, this study demonstrated that SSPR therapy, whether used alone or in combination with SOC, conferred significant clinical benefits in treating RD. These benefits were reflected in lower DHI and VAS scores, reduced TCM syndrome scores and the recurrence rate, and higher dizziness resolution rate and clinical effective rate. SSPR therapy was also superior to both no-intervention and placebo in reducing DHI and VAS scores. DHI, scored on a continuous scale from 0 to 100, represents a recognized standard in clinical practice for assessing multi-dimensional vestibular handicaps and quality of life (15, 16). Therefore, it served as the primary efficacy measure for outcome evaluation in patients with RD. A previous study established that a score reduction of at least 18 points represents the minimum clinically important difference (MCID) to indicate a meaningful change (15), whereas another study (57) subsequently determined via ROC curve analysis that an 11-point reduction represents the threshold perceived by patients. Our meta-analysis showed that SSPR therapy consistently reduced DHI scores by approximately 9 to 11 points, whether as monotherapy (vs. placebo or SOC) or combined with SOC (vs. SOC), approaching the patient-perceived threshold. SSPR therapy substituted for central psychotropic-containing regimens achieved an 11.35-point reduction, while SSPR therapy combined with VRT and WM yielded a 20.10-point reduction over VRT plus WM. In clinical practice, conventional treatments or natural recovery often lead to partial alleviation of dizziness. Importantly, our findings demonstrate that SSPR therapy may offer patients meaningful further clinical improvement.

In terms of safety, no significant difference in adverse event rate was observed between the experimental group and the control group, suggesting no additional adverse event risk associated with SSPR therapy. Nevertheless, because the evidence was constrained by incomplete reporting, a potential risk of underreporting, and low event counts across the included studies, the present evidence remains insufficient to establish its definitive safety profile. Additionally, under different control group, some outcomes exhibited a certain degree of heterogeneity and limitations. SSPR therapy showed no significant difference in dizziness resolution rate, TCM syndrome score and clinical effective rate compared to no-intervention and also showed no significant difference in dizziness resolution rate compared with placebo. The observed results may be related to the limited sample size or low number of events, and variations in the sensitivity of different outcome measures to clinical efficacy.

Encouragingly, the direction of treatment effect was highly consistent across all subgroups and individual comparison categories defined by different SOC regimens, supporting the robustness of these findings. Specifically, when used as a substitute, SSPR therapy demonstrated superior clinical benefits compared with individual SOC options, including vestibular monotherapy and central psychotropic-containing regimens. Similarly, as an integrative strategy, SSPR combined with SOC outperformed SOC alone, regardless of whether the baseline regimen involved Western medicine or VRT. Additionally, while single-study estimates for other comparators, such as phytopharmaceutical monotherapy and VRT, also favored SSPR therapy, these results require further validation. Collectively, these findings hold meaningful clinical implications. For refractory patients who respond poorly to or have poor adherence to conventional regimens, SSPR therapy may serve as a valuable alternative or adjunct treatment.

Although our inclusion criteria explicitly restricted the intervention to SSPR prescriptions, marked heterogeneity in specific herbal components and treatment duration remains substantial. To further explore these sources of variation, subgroup analyses were conducted. Regarding treatment duration, while overall therapeutic effect remained relatively robust across different treatment durations, extending the intervention beyond 14 days (specifically 28 days in all included studies) reduced heterogeneity. This suggests that longer treatment regimens may stabilize therapeutic outcomes and yield more consistent effect estimates across studies. However, the number of studies in the 28-day subgroup remains limited, and high heterogeneity persisted in shorter-duration subgroups, demonstrating that treatment duration is not the sole source of the observed heterogeneity. Subgroup analysis demonstrated that SSPR prescriptions containing ≥ 10 herbs yielded more consistent effect estimates with reduced heterogeneity. This indicates that variations in the number of herbal components contribute to observed heterogeneity. However, from a TCM clinical perspective, this finding does not imply that adding more herbs guarantees superior efficacy. Prescriptions with a relatively higher herb count are usually based on a core principle, with additional herbal components that target common accompanying symptoms or secondary pathogenesis. Given the complex clinical presentations frequently encountered in clinical practice, such expanded prescriptions may yield more stable therapeutic effects. In contrast, simpler prescriptions, mainly target the primary pathogenesis with limited management of accompanying symptoms, which may lead to varying therapeutic effects among different patients and consequently increase heterogeneity.

### 4.2 TCM pathogenesis and herbal prescription regularity

Based on TCM theory, spleen deficiency and damp phlegm are considered the pathogenesis of RD. Although the otolith particles are successfully restored to their original positions by CRPs, impaired spleen transportation and unresolved phlegm-turbidity persist in patients owing to irregular diet, emotional disturbances, advanced age, constitutional weakness and other factors, thereby causing persistent dizziness. Zhu Danxi proposed, “The method of treating phlegm lies in strengthening the spleen earth and drying the spleen dampness, which treats the disease from its root.” SSPR therapy exerts its therapeutic effects by activating and fortifying the middle jiao to eliminate the source of phlegm generation, and resolving existing phlegm accumulation. Guided by the principles of SSPR therapy, the application of TCM herbal formulas for RD perfectly exemplifies the unique holistic regulatory superiority of TCM. From a modern medical perspective, it is hypothesized that SSPR therapy exert its therapeutic effects through the synergistic intervention of multi-component, multi-target, and multi-pathway networks. It is plausible that SSPR therapy may facilitate the relief of dizziness symptoms and contribute to restoring vestibular homeostasis by mitigating oxidative stress, correcting hemorheological disorders, and modulating inner ear microcirculation. Simultaneously, from the TCM perspective, this therapy regulates the functions of the spleen and stomach, eliminates the internal retained phlegm-dampness, and rectifies systemic disharmony. With clear yang ascending and turbid yin descending, the qi mechanism flows smoothly, which may potentially facilitate symptomatic recovery and reduce the risk of symptom recurrence.

The 37 included trials involved a total of 69 Chinese herbs. Regarding the medicinal nature of herbs, warm-natured herbs demonstrated the highest frequency of occurrence (182 times), followed by neutral (115 times), cold (71 times), cool (15 times), and hot (3 times) herbs. The overwhelming prevalence of warm properties highly conforms to the fundamental therapeutic rationale of SSPR therapy in treating RD. Specifically, warm herbs are employed to activate yang-qi and assist the spleen transportation, thereby naturally eliminating phlegm-turbidity. As stated in *Synopsis of Prescriptions of the Golden Chamber*, “Phlegm-fluid disorders ought to be harmonized using warm herbs.” According to our herbal frequency statistics, Pinelliae Rhizoma (*Banxia*) ranked first with 34 occurrences, primarily utilized for its effects in drying dampness and resolving phlegm. Glycyrrhizae Radix et Rhizoma (*Gancao*, 31 times) acts to tonify the middle jiao and resolve phlegm. Atractylodis Macrocephalae Rhizoma (*Baizhu*, 31 times) serves to replenish qi, strengthen spleen and dry dampness. Poria (*Fuling*, 30 times) functions to promote diuresis to eliminate dampness, fortify spleen and invigorate the spleen and calm the heart. Citri Reticulatae Pericarpium (*Chenpi*) and Citri Grandis Exocarpium (*Juhong*) appeared 30 times, both exerting the efficacy of regulating qi, drying dampness, and resolving phlegm. As the celebrated physician Li Dongyuan asserted, “Retaining the white spongy layer tonifies the spleen and stomach, removing it regulates lung qi.” Gastrodiae Rhizoma (*Tianma*, 28 times) extinguishing wind without injuring vital qi, is exceptionally good at calming the liver, dispelling wind, and unblocking collaterals.

Previous studies have identified that *Fuling-Baizhu* drug pair is frequently utilized in clinical practice to treat vertigo. Evidence from network pharmacology indicates that this drug pair may effect via pathways such as the calcium signaling pathway and the estrogen signaling pathway to modulate inner ear microcirculation and mitigate the risk of otolith dislocation (58). Total alkaloids from *Banxia* can inhibit the hyperexcitability of neurons via *γ*-aminobutyric acid (GABA) regulation, and also exert anxiolytic effects. The n-butyl alcohol extract of *Banxia* alleviates oxidative stress and restore the vestibular microenvironment (59). *Tianma* is one of the most commonly used Chinese herbs besides spleen-strengthening herbs and phlegm-resolving herbs. Studies have shown that the active components of *Tianma* exert potent anti-vertigo, antidepressant, and circulatory modulation effects by elevating GABA levels (60). Furthermore, they significantly enhance behavioral recovery in vertigo models, alleviate vestibular locomotor dysfunction, and promote vestibular compensation (60–62).

Remarkably, the top five individual herbs (*Banxia*, *Gancao*, *Baizhu*, *Fuling*, *Tianma*) derived from our study ranked by usage frequency are exactly the key components comprising BBTD. BBTD serves as the foundational formula for resolving phlegm, extinguishing wind, strengthening spleen, and dispelling dampness, which is widely utilized in clinical practice to treat dizziness induced by multiple etiologies (63). Internal obstruction of phlegm-turbidity corresponds to abnormal hemorheology in Western medicine. Studies have demonstrated that BBTD markedly correct hemorheological disorders by regulating blood lipids as well as vasoactive factors endothelin-1 (ET-1) and calcitonin gene related peptide (CGRP), thereby improving inner ear circulation and alleviating dizziness (11, 64). Despite spleen deficiency and phlegm-dampness being the primary pathogenesis of RD, single syndrome patterns are rarely encountered in clinical practice. Treatments should be formulated holistically to tackle concurrent syndrome patterns.

### 4.3 Strengths and limitations

We performed separate meta-analyses across four distinct types of comparison groups. In terms of outcomes, our evaluation primarily focused on DHI score, dizziness resolution rate, VAS score, which represent the improvement of patients' dizziness symptoms. Furthermore, by evaluating recurrence rate, we assessed the sustained efficacy of SSPR therapy based on the available and variable follow-up periods. To explore potential sources of substantial heterogeneity, subgroup analyses were conducted based on specific types of SOC, number of herbal components and treatment duration, and sensitivity analyses were further performed to evaluate the robustness of the findings. By comparing with diverse SOC, this study comprehensively evaluated the therapeutic potential and clinical prospects of SSPR therapy. Additionally, we extracted the SSPR prescriptions from the included studies and ranked the utilization frequency of individual Chinese herbs, aiming to provide guidance for the clinical application of SSPR therapy. Notably, we acknowledge that the outcomes in this study are inherently derived from patient-reported evaluations. This is largely unavoidable, as RD is a subjective symptom and validated objective biomarkers remain unavailable in routine clinical practice. To reduce the methodological variance and potential bias in subjective assessment, “dizziness resolution rate” was set as a binary extreme endpoint, while for “clinical effective rate”, we excluded studies that relied solely on self-reported symptom relief or lacked clear evaluation criteria, retaining only trials that used relative score reduction from DHI or TCM syndrome scales. Although minor variations exist in the reduction thresholds across trials, and specific TCM syndrome scales varied, these outcomes consistently reflect meaningful clinical improvement, ensuring acceptable comparability and enhancing the robustness of the pooled results.

Several limitations in this study should be acknowledged. Firstly, all the included trials were conducted in China, which may limit the generalizability of our findings to global populations with different ethnic backgrounds. Secondly, some studies failed to specify allocation concealment and blinding; moreover, none of the literature reporting dropouts utilized Intention-to-Treat (ITT) analysis to account for missing data, leading to a high risk of bias in a portion of the included trials. Thirdly, only seven studies reported recurrence rate, and long-term follow-up data are insufficient, making it difficult to evaluate the sustained clinical benefits after treatment. In addition, potential publication bias was limited to specific outcomes, including TCM syndrome score and clinical effective rate, which may stem from differences in outcome evaluation criteria (such as inconsistent definitions of clinical effectiveness), variation in assessment instruments (such as TCM syndrome scales) and the inherent subjectivity of these evaluations across the included trials. Moreover, the outcome measures in the included studies predominantly relied on subjective scales, lacking objective parameters. Considering the highly subjective nature of RD symptoms, future trials need to adopt additional objective quantitative evaluation tools.

## 5 Conclusion

Low-certainty evidence suggests that SSPR therapy whether used alone or combined with SOC, demonstrates potential efficacy in alleviating dizziness and related clinical symptoms in RD patients, and may reduce the recurrence rate, with insufficient evidence to determine safety. However, due to the low methodological quality of the included studies, these findings require further validation through future high-quality clinical trials.

## Data Availability

Publicly available datasets were analyzed in this study. This data can be found here: All data were extracted from published studies. The extracted baseline characteristics, intervention details (including Chinese herbal formula composition, preparation, source, administration), and summary effect sizes are provided in Table 1, Supplementary Tables S3, S5, S6, S9. No public database was used.
