# Supplementary material for: Efficacy and safety of spleen-strengthening and phlegm-resolving herbal therapy for residual dizziness after repositioning of benign paroxysmal positional vertigo: a systematic review and meta-analysis of randomized controlled trials
Source: Front Med (Lausanne). 2026 Oct 19;13:1949948. doi: 10.3389/fmed.2026.1949948 (PMC13724474; doi:10.3389/fmed.2026.1949948)
Supplement: Supplementary file 2 [file Table1.docx]

**Supplementary Table 1** PRISMA 2020 Checklist.

| **Section and Topic** | **Item #** | **Checklist item** | **Location where item is reported** |
| --- | --- | --- | --- |
| **TITLE** | | |  |
| Title | 1 | Identify the report as a systematic review. | Title |
| **ABSTRACT** | | |  |
| Abstract | 2 | See the PRISMA 2020 for Abstracts checklist. | Abstract |
| **INTRODUCTION** | | |  |
| Rationale | 3 | Describe the rationale for the review in the context of existing knowledge. | Introduction |
| Objectives | 4 | Provide an explicit statement of the objective(s) or question(s) the review addresses. | Introduction |
| **METHODS** | | |  |
| Eligibility criteria | 5 | Specify the inclusion and exclusion criteria for the review and how studies were grouped for the syntheses. | 2.2 |
| Information sources | 6 | Specify all databases, registers, websites, organisations, reference lists and other sources searched or consulted to identify studies. Specify the date when each source was last searched or consulted. | 2.3 |
| Search strategy | 7 | Present the full search strategies for all databases, registers and websites, including any filters and limits used. | Supplementary Table 2 |
| Selection process | 8 | Specify the methods used to decide whether a study met the inclusion criteria of the review, including how many reviewers screened each record and each report retrieved, whether they worked independently, and if applicable, details of automation tools used in the process. | 2.4 |
| Data collection process | 9 | Specify the methods used to collect data from reports, including how many reviewers collected data from each report, whether they worked independently, any processes for obtaining or confirming data from study investigators, and if applicable, details of automation tools used in the process. | 2.4 |
| Data items | 10a | List and define all outcomes for which data were sought. Specify whether all results that were compatible with each outcome domain in each study were sought (e.g. for all measures, time points, analyses), and if not, the methods used to decide which results to collect. | 2.4 |
|  | 10b | List and define all other variables for which data were sought (e.g. participant and intervention characteristics, funding sources). Describe any assumptions made about any missing or unclear information. | 2.4 |
| Study risk of bias assessment | 11 | Specify the methods used to assess risk of bias in the included studies, including details of the tool(s) used, how many reviewers assessed each study and whether they worked independently, and if applicable, details of automation tools used in the process. | 2.5 |
| Effect measures | 12 | Specify for each outcome the effect measure(s) (e.g. risk ratio, mean difference) used in the synthesis or presentation of results. | 2.6 |
| Synthesis methods | 13a | Describe the processes used to decide which studies were eligible for each synthesis (e.g. tabulating the study intervention characteristics and comparing against the planned groups for each synthesis (item #5)). | 2.6 |
|  | 13b | Describe any methods required to prepare the data for presentation or synthesis, such as handling of missing summary statistics, or data conversions. | 2.6 |
|  | 13c | Describe any methods used to tabulate or visually display results of individual studies and syntheses. | 2.6 |
|  | 13d | Describe any methods used to synthesize results and provide a rationale for the choice(s). If meta-analysis was performed, describe the model(s), method(s) to identify the presence and extent of statistical heterogeneity, and software package(s) used. | 2.6 |
|  | 13e | Describe any methods used to explore possible causes of heterogeneity among study results (e.g. subgroup analysis, meta-regression). | 2.6 |
|  | 13f | Describe any sensitivity analyses conducted to assess robustness of the synthesized results. | 2.6 |
| Reporting bias assessment | 14 | Describe any methods used to assess risk of bias due to missing results in a synthesis (arising from reporting biases). | 2.6 |
| Certainty assessment | 15 | Describe any methods used to assess certainty (or confidence) in the body of evidence for an outcome. | 2.6 |
| **RESULTS** | | |  |
| Study selection | 16a | Describe the results of the search and selection process, from the number of records identified in the search to the number of studies included in the review, ideally using a flow diagram. | Figure 1 |
|  | 16b | Cite studies that might appear to meet the inclusion criteria, but which were excluded, and explain why they were excluded. | Figure 1 |
| Study characteristics | 17 | Cite each included study and present its characteristics. | Table 1,  Table 2,  Supplementary Table 3 |
| Risk of bias in studies | 18 | Present assessments of risk of bias for each included study. | Figure 2, Supplementary Table 4 |
| Results of individual studies | 19 | For all outcomes, present, for each study: (a) summary statistics for each group (where appropriate) and (b) an effect estimate and its precision (e.g. confidence/credible interval), ideally using structured tables or plots. | Supplementary Table 6, Supplementary Table 7 |
| Results of syntheses | 20a | For each synthesis, briefly summarise the characteristics and risk of bias among contributing studies. | 3.2, 3.3 |
|  | 20b | Present results of all statistical syntheses conducted. If meta-analysis was done, present for each the summary estimate and its precision (e.g. confidence/credible interval) and measures of statistical heterogeneity. If comparing groups, describe the direction of the effect. | 3.4,  Supplementary Table 6, Supplementary Table 7 |
|  | 20c | Present results of all investigations of possible causes of heterogeneity among study results. | 3.5 |
|  | 20d | Present results of all sensitivity analyses conducted to assess the robustness of the synthesized results. | 3.6 |
| Reporting biases | 21 | Present assessments of risk of bias due to missing results (arising from reporting biases) for each synthesis assessed. | 3.7 |
| Certainty of evidence | 22 | Present assessments of certainty (or confidence) in the body of evidence for each outcome assessed. | 3.8, Supplementary Table 8 |
| **DISCUSSION** | | |  |
| Discussion | 23a | Provide a general interpretation of the results in the context of other evidence. | 4.1 |
|  | 23b | Discuss any limitations of the evidence included in the review. | 4.3 |
|  | 23c | Discuss any limitations of the review processes used. | 4.3 |
|  | 23d | Discuss implications of the results for practice, policy, and future research. | 4.2 |
| **OTHER INFORMATION** | | |  |
| Registration and protocol | 24a | Provide registration information for the review, including register name and registration number, or state that the review was not registered. | 2.1 |
|  | 24b | Indicate where the review protocol can be accessed, or state that a protocol was not prepared. | 2.1 |
|  | 24c | Describe and explain any amendments to information provided at registration or in the protocol. | 2.1 |
| Support | 25 | Describe sources of financial or non-financial support for the review, and the role of the funders or sponsors in the review. | Title page |
| Competing interests | 26 | Declare any competing interests of review authors. | Conflict of Interest |
| Availability of data, code and other materials | 27 | Report which of the following are publicly available and where they can be found: template data collection forms; data extracted from included studies; data used for all analyses; analytic code; any other materials used in the review. | Data availability |

**Supplementary Table 2** Searching strategies.

| Database searched | Records |
| --- | --- |
| Chinese National Knowledge Infrastructure (CNKI) | 506 |
| Wanfang Database | 582 |
| Chinese Science and Technology Journals Database (VIP) | 822 |
| Chinese Biomedical Database (Sinomed) | 450 |
| PubMed | 7 |
| Web of Science (WOS) | 4 |
| Cochrane library | 11 |
| Embase | 55 |
| Total | 2437 |

**CNKI search strategy**

SU%=（健脾+化痰+祛痰+中药+中医药+汤+颗粒+中医+中成药） AND SU%= （良性阵发性位置性眩晕+BPPV+耳石症）

**Wanfang Database search strategy**

（主题:（健脾）or 主题:(化痰) or 主题:(祛痰) or 主题:(中药) or 主题:(中医药)or 主题:(汤) or 主题:(颗粒) or 主题:(中医) or 主题:(中成药)） and（主题:良性阵发性位置性眩晕 or 主题:耳石症 or 主题:BPPV）

**VIP search strategy**

（U=健脾 OR U=化痰 OR U=祛痰 OR U=中药 OR U= 中医药 OR U=颗粒 OR U=汤 OR U=中医 OR U=中成药） AND （M=良性阵发性位置性眩晕 OR M=耳石症 OR M=BPPV）

**SinoMed search strategy**

("健脾"[常用字段:智能] OR "化痰"[常用字段:智能] OR "祛痰"[常用字段:智能] OR "中药"[常用字段:智能] OR "中医药"[常用字段:智能] OR "汤"[常用字段:智能] OR "颗粒"[常用字段:智能] OR "中医"[常用字段:智能] OR "中成药"[常用字段:智能]) AND （"BPPV"[常用字段:智能] OR "良性阵发性位置性眩晕"[常用字段:智能] OR "耳石症"[常用字段:智能]）

**Pubmed search strategy**

**((((((((((((("Drugs, Chinese Herbal"[MeSH Terms]) OR ("Medicine, Chinese Traditional"[MeSH Terms])) OR ("traditional Chinese medicine"[Title/Abstract])) OR ("Chinese medicine"[Title/Abstract])) OR ("traditional medicine"[Title/Abstract])) OR ("herbal medicine"[Title/Abstract])) OR ("herbal therapy"[Title/Abstract])) OR ("herbal remedy"[Title/Abstract])) OR ("herbal drug"[Title/Abstract])) OR ("Chinese Herbal Formula"[Title/Abstract])) OR ("Chinese Herb"[Title/Abstract])) OR (Phytotherapy[MeSH Terms])) OR ("Chinese drug"[Title/Abstract])) AND ((((("benign paroxysmal positional vertigo"[MeSH Terms]) OR ("Benign Recurrent Vertigo*"[Title/Abstract])) OR ("Familial Vestibulopath*"[Title/Abstract])) OR ("Benign Paroxysmal Positional"[Title/Abstract])) OR (BPPV[Title/Abstract]))**

****WOS search strategy****

| **Number** | **Search terms** |
| --- | --- |
| #1 | **("Drugs, Chinese Herbal"** (Topic) or **"Medicine, Chinese Traditional"** (Topic) or **"traditional Chinese medicine"** (Topic) or **"Chinese medicine"** (Topic) or **"traditional medicine"** (Topic) or **"herbal medicine"** (Topic) or **"herbal therapy"** (Topic) or **"herbal remedy"** (Topic) or **"herbal drug"** (Topic) or **"Chinese Herbal Formula"** (Topic) or **"Chinese Herb"** (Topic) or **Phytotherapy** (Topic) or **"Chinese drug"** (Topic) |
| #2 | (**"benign paroxysmal positional vertigo"** (Topic) or **"Benign Recurrent Vertigo*"** (Topic) or **"Familial Vestibulopath*"** (Topic) or **"Benign Paroxysmal Positional"** (Topic) or **BPPV** (Topic)) |
| #3 | **(**#1) AND #2 |

****Cochrane library search strategy****

| **Number** | **Search terms** |
| --- | --- |
| #1 | **MeSH descriptor: [Medicine, Chinese Traditional] explode all trees** |
| #2 | **( "traditional Chinese medicine" ):ti,ab,kw OR ("Chinese medicine"):ti,ab,kw OR ("traditional medicine"):ti,ab,kw OR ("herbal medicine"):ti,ab,kw OR ("herbal therapy"):ti,ab,kw OR ("herbal remedy"):ti,ab,kw OR ("herbal drug"):ti,ab,kw OR ("Chinese Herbal Formula"):ti,ab,kw OR ("Chinese Herb"):ti,ab,kw OR ("Chinese drug")**:ti,ab,kw |
| #3 | **MeSH descriptor: [Phytotherapy] explode all trees** |
| #4 | #1 OR #2 OR #3 |
| #5 | **MeSH descriptor: [Benign Paroxysmal Positional Vertigo] explode all trees** |
| #6 | ("Benign Recurrent Vertigo" ):ti,ab,kw OR ("Familial Vestibulopathy" ):ti,ab,kw OR ("Benign Paroxysmal Positional" ):ti,ab,kw OR (BPPV):ti,ab,kw OR (Familial Vestibulopathies):ti,ab,kw |
| #7 | #5 OR #6 |
| #8 | #4 AND #7 |

****Embase search strategy****

| **Number** | **Search terms** |
| --- | --- |
| #1 | ‘benign paroxysmal positional vertigo’/exp OR ‘benign paroxysmal positional vertigo’ |
| #2 | ‘paroxysmal positional vertigo‘:ab,kw,ti OR ‘familial benign recurrent vertigo‘:ab,kw,ti OR ‘BPPV‘:ab,kw,ti OR ‘benign paroxysmal postural vertigo‘:ab,kw,ti OR ‘benign postural paroxysmal vertigo‘:ab,kw,ti OR ‘familial vestibulopathy‘:ab,kw,ti OR ‘positional paroxysmal vertigo’:ab,kw,ti |
| #3 | #1 OR #2 |
| #4 | ‘herbal medicine’/exp OR ‘herbal medicine’ |
| #5 | ‘phytotherapy’/exp OR ‘phytotherapy’ |
| #6 | ‘herbal medicine’/exp OR ‘herbal medicine’ |
| #7 | ‘Chinese herbal medicine‘:ab,kw,ti OR ‘traditional medicine‘:ab,kw,ti OR ‘traditional Chinese medicine‘:ab,kw,ti OR ‘medicine, Chinese traditional‘:ab,kw,ti OR ‘**herbal therapy**‘:ab,kw,ti OR ‘**herbal remedy**‘:ab,kw,ti OR ‘**herbal drug**‘:ab,kw,ti OR ‘**Chinese Herbal Formula**‘:ab,kw,ti OR ‘**Chinese Herb**‘:ab,kw,ti OR ‘**Chinese drug**‘:ab,kw,ti |
| #8 | #4 OR #5 OR #6 OR #7 |
| #9 | #3 AND #8 |

**Supplementary Table 3** Specific ingredients and dosages of SSPR prescriptions.

| **Study ID** | **Name of prescriptions** | **Compositions** | **Preparation and source** | **Administration and dosage** |
| --- | --- | --- | --- | --- |
| **SSPR therapy versus no intervention** | | |  |  |
| Fan SH 2020 | Pingxuan Dingyun Decoction | Pinelliae Rhizoma (Banxia) 10g, Gastrodiae Rhizoma (Tianma) 10g, Poria (Fuling) 10g, Atractylodis Macrocephalae Rhizoma (Baizhu) 10g, Rehmanniae Radix Preparata (Shudihuang) 10g, Angelica sinensis (Oliv.) Diels (Danggui) 12g, Citri Reticulatae Pericarpium (Chenpi) 10g, Drynariae Rhizoma (Gusuibu) 10g, Bombyx Batryticatus (Jiangcan) 10g, Cicadae Periostracum (Chantui) 10g, Tribuli Fructus (Jili) 15g, Viticis Fructus (Manjingzi) 10g, Scorpio (Quanxie) 3g, Glycyrrhizae Radix et Rhizoma (Gancao) 6g. | Centrally decocted by the Pharmacy Department of the Affiliated Hospital of Hunan Institute of TCM. Decocted in water to 200 mL. | One dose daily, taken in two divided doses. |
| Liu J 2022 | TCM | Pinelliae Rhizoma (Banxia) 9g, Gastrodiae Rhizoma (Tianma) 15g, Atractylodis Macrocephalae Rhizoma (Baizhu) 30g, Citri Reticulatae Pericarpium (Chenpi) 15g, Poria (Fuling) 15g, Glycyrrhizae Radix et Rhizoma (Gancao) 6g, Zingiberis Rhizoma Recens (Shengjiang) 6g, Jujubae Fructus (Dazao) 6g, Acori Tatarinowii Rhizoma (Shichangpu) 15g, Rhizoma Alismatis (Zexie) 10g. | Sourced from the Pharmacy Department of Changping Hospital of TCM. Concentrately decocted in water to 200 mL. | Taken in two 100 mL doses (morning and evening). |
| Shu F 2022 | Xuandekang Granule | Rhizoma Alismatis (Zexie) 30g, Atractylodis Macrocephalae Rhizoma (Baizhu) 20g, Pinelliae Rhizoma (Banxia) 10g, Poria (Fuling) 25g, Gastrodiae Rhizoma (Tianma) 15g, Uncariae Ramulus cum Uncis (Gouteng) 25g, Citri Reticulatae Pericarpium (Chenpi) 15g, Glycyrrhizae Radix et Rhizoma (Gancao) 15g. | Sourced from the Outpatient Pharmacy of the Affiliated Hospital of Liaoning University of TCM. | Taken twice daily (30 min post-meal in the morning and evening), 1 packet per time, dissolved in warm boiled water. |
| ZhangY 2021 | Huatan Tongnao Decoction | Gastrodiae Rhizoma (Tianma) 10g, Atractylodis Macrocephalae Rhizoma (Baizhu) 10g, Pinelliae Rhizoma (Banxia) 10 g, Bombyx Batryticatus (Jiangcan) 10g, Citri Reticulatae Pericarpium (Chenpi) 10g, Poria (Fuling) 10g, Acori Tatarinowii Rhizoma (Shichangpu) 10g, Polygalae Radix (Yuanzhi) 10g, Viticis Fructus (Manjingzi) 10g, Salviae Miltiorrhizae Radix et Rhizoma (Danshen) 30g, Glycyrrhizae Radix et Rhizoma (Gancao) 5g. | Chinese herbal formula granules (manufactured by Guangdong Yifang Pharmaceutical Co., Ltd.). | One dose daily, dissolved in 300 mL of boiling water, and taken warm in two divided doses. |

**Supplementary Table 3** Specific ingredients and dosages of SSPR prescriptions (Continued).

| **Study ID** | **Name of prescriptions** | **Compositions** | **Preparation and source** | **Administration and dosage** |
| --- | --- | --- | --- | --- |
| **SSPR therapy versus placebo** | | | | |
| Liu S 2025 | Tianma Jiannao Granule+VRT | Pinelliae Rhizoma (Banxia) 9g, Atractylodis Macrocephalae Rhizoma (Baizhu) 10g, Gastrodiae Rhizoma (Tianma) 10g, Uncariae Ramulus cum Uncis (Gouteng) 10g, Rhodiolae Crenulatae Radix et Rhizoma (Hongjingtian) 6g, Poria (Fuling) 15g, Lycopi Herba (Zelan) 10g, Acori Tatarinowii Rhizoma (Shichangpu) 10g. | Hospital preparation of Dongguan TCM Hospital (10 g/sachet, Approval No. Z20190025000). | Taken three times daily, 10g per time, 30 min post-meal. |
| Wu WL 2023 | Tongqiao Jingxuan Decoction | Bupleuri Radix (Chaihu)10g, Cyperi Rhizoma (Xiangfu) 10g, Chuanxiong Rhizoma (Chuanxiong) 10g, Rhizoma Alismatis (Zexie) 15g, Atractylodis Macrocephalae Rhizoma (Baizhu) 10g, Acori Tatarinowii Rhizoma (Shichangpu) 10g, Liquidambaris Fructus (Lulutong) 10g, Uncariae Ramulus cum Uncis (Gouteng) 10g, Margaritifera Concha (Zhenzhumu) 30g, Glycyrrhizae Radix et Rhizoma (Gancao) 6g. | Chinese herbal formula granules (standardized production by Beijing Kangrentang Pharmaceutical Co., Ltd.). | Taken twice daily, 1 sachet per time, dissolved in 50 - 150 mL of warm boiled water. |
| Zhu YJ 2021 | Jianpi Huayin Decoction | Poria (Fuling) 30g , Atractylodis Macrocephalae Rhizoma (Baizhu) 10g, Rhizoma Alismatis (Zexie) 15g, Astragali Radix (Huangqi) 30g, Cinnamomi Ramulus (Guizhi) 10g, Acori Tatarinowii Rhizoma (Shichangpu) 10g, Curcumae Radix (Yujin) 10g, Liquidambaris Fructus (Lulutong) 15g, Bupleuri Radix (Chaihu) 10g, Achyranthis Bidentatae Radix (Niuxi) 10g, Chrysanthemi Flos (Juhua) 10g, Glycyrrhizae Radix et Rhizoma (Gancao) 6g. | Not reported | Taken twice daily (30 min post-meal in the morning and evening), 1 sachet per time. |
| **SSPR therapy versus SOC** | | | | |
| Cai HC 2016 | Modified Xiaqi Decoction | Poria (Fuling) 15g, Polygoni Multiflori Radix Preparata (Heshouwu) 20g, Moutan Cortex (Mudanpi) 10g, Paeoniae Radix Alba (Baishao) 15g, Citri Grandis Exocarpium (Juhong) 10g, Pinelliae Rhizoma (Banxia) 10g, Armeniacae Semen Amarum (Kuxingren) 10g, Glycyrrhizae Radix et Rhizoma (Gancao) 5g. | Centrally decocted by Futian District Hospital of TCM, Shenzhen. | One dose daily, decocted in water, taken in two divided doses. |

**Supplementary Table 3** Specific ingredients and dosages of SSPR prescriptions (Continued).

| **Study ID** | **Name of prescriptions** | **Compositions** | **Preparation and source** | **Administration and dosage** |
| --- | --- | --- | --- | --- |
| Huang SH 2020 | Zhiyun Huatan Decoction | Pinelliae Rhizoma (Banxia) 10g, Gastrodiae Rhizoma (Tianma) 10g, Poria (Fuling) 15g, Atractylodis Macrocephalae Rhizoma (Baizhu) 10g, Citri Reticulatae Pericarpium (Chenpi) 10g, Chuanxiong Rhizoma (Chuanxiong) 16g, Polygalae Radix (Yuanzhi) 10g, Acori Tatarinowii Rhizoma (Shichangpu) 10g, Uncariae Ramulus cum Uncis (Gouteng) 15g, Rhodiolae Crenulatae Radix et Rhizoma (Hongjingtian) 15g, Siegesbeckiae Herba (Xixiancao) 15g, Glycyrrhizae Radix et Rhizoma (Gancao) 6g. | Sourced from the Outpatient Pharmacy of Xiamen Hospital of TCM (produced by Kangmei Pharmaceutical Co., Ltd.), centrally decocted by the Decoction Room using an automatic decoction machine into two 150 mL vacuum-sealed bags (300 mL per dose in total). | Taken twice daily (30 min post-meal in the morning and evening), 1 sachet per time. |
| Huang ST 2021 | Modified BBTD | Pinelliae Rhizoma (Banxia) 12g, Gastrodiae Rhizoma (Tianma) 12g, Poria (Fuling) 20g, Citri Grandis Exocarpium (Juhong) 10g, Atractylodis Macrocephalae Rhizoma (Baizhu) 10g, Glycyrrhizae Radix et Rhizoma (Gancao) 6g, Jujubae Fructus (Dazao) 6g, Zingiberis Rhizoma Recens (Shengjiang) 9g. | Not reported | Boiled and simmered over low heat to about 400 mL, taken twice daily (morning and evening), 200 mL per dose. |
| Huang Y 2017 | Modified Wendan Decoction | Pinelliae Rhizoma (Banxia) 15g, Bambusae Caulis in Taeniam (Zhuru) 12g, Aurantii Fructus Immaturus (Zhishi) 12g, Anemarrhenae Rhizoma (Zhimu) 10g, Citri Reticulatae Pericarpium (Chenpi) 6g, Gypsum Fibrosum (Shigao) 6g, Glycyrrhizae Radix et Rhizoma (Gancao) 6g. | Not reported | One dose daily, decocted in water to 100 mL and taken twice daily (morning and evening). |

**Supplementary Table 3** Specific ingredients and dosages of SSPR prescriptions (Continued).

| **Study ID** | **Name of prescriptions** | **Compositions** | **Preparation and source** | **Administration and dosage** |
| --- | --- | --- | --- | --- |
| Lin XQ 2019 | Modified BBTD | Pinelliae Rhizoma (Banxia) 12g, Gastrodiae Rhizoma (Tianma) 12g, Atractylodis Macrocephalae Rhizoma (Baizhu) 15g, Poria (Fuling) 24g, Citri Reticulatae Pericarpium (Chenpi) 10g, Bambusae Caulis in Taeniam (Zhuru) 12g, Aurantii Fructus Immaturus (Zhishi) 12g, Rhizoma Alismatis (Zexie) 10g, Acori Tatarinowii Rhizoma (Shichangpu) 9g, Zingiberis Rhizoma Recens (Shengjiang) 6g, Jujubae Fructus (Dazao) 6g, Glycyrrhizae Radix et Rhizoma (Gancao) 3g. | Centrally decocted by the Pharmacy of the Second People's Hospital Affiliated to Fujian University of TCM (electric decoction machine: manufactured by Beijing Donghuayuan, Model No. YJD20-GL). | One dose daily, taken warm twice daily (30 min post-meal in the morning and evening). |
| Liu YF 2026 | Qingqiao Dingxuan Decoction | Chuanxiong Rhizoma (Chuanxiong) 20g, Rhizoma Alismatis (Zexie) 15g, Gastrodiae Rhizoma (Tianma) 12g, Atractylodis Macrocephalae Rhizoma (Baizhu) 10g, Chrysanthemi Flos (Juhua) 20g, Acori Tatarinowii Rhizoma (Shichangpu) 10g, Pinelliae Rhizoma (Banxia) 10g, Bombyx Batryticatus (Jiangcan) 10g, Poria (Fuling) 10g, Scorpio (Quanxie) 3g, Os Draconis (Longgu) 30g, Ostreae Concha (Muli) 30g, Salviae Miltiorrhizae Radix et Rhizoma (Danshen) 15g. | Sources from the Pharmacy of the Affiliated Hospital of Changzhi Institute of TCM, Shanxi Province, decocted in water to 200 mL. | Taken warm twice daily (morning and evening on an empty stomach). |
| Qin ZG 2020 | Modified BBTD combined with Taohong Siwu Decoction | Pinelliae Rhizoma (Banxia) 12g, Atractylodis Macrocephalae Rhizoma (Baizhu) 12g, Gastrodiae Rhizoma (Tianma) 10g, Poria (Fuling) 15g, Citri Reticulatae Pericarpium (Chenpi) 6g, Rhizoma Alismatis (Zexie) 10g, Jujubae Fructus (Dazao) 3 pieces, Glycyrrhizae Radix et Rhizoma (Gancao) 6g, Persicae Semen (Taoren) 10g, Chuanxiong Rhizoma (Chuanxiong) 6g, Salviae Miltiorrhizae Radix et Rhizoma (Danshen) 15g, Curcumae Radix (Yujin) 10g. | Not reported | One dose daily, decocted in water to 150 mL and taken 3 times daily, 50 mL per time. |

**Supplementary Table 3** Specific ingredients and dosages of SSPR prescriptions (Continued).

| **Study ID** | **Name of prescriptions** | **Compositions** | **Preparation and source** | **Administration and dosage** |
| --- | --- | --- | --- | --- |
| Shao DF 2023 | Wenzhong Dingxuan Decoction | Zingiberis Rhizoma (Ganjiang) 9g, Citri Reticulatae Pericarpium (Chenpi) 10g, Codonopsis Radix（Dangshen) 10g, Rhizoma Alismatis (Zexie) 10g, Lycopi Herba (Zelan) 10g, Poria (Fuling) 30g, Atractylodis Macrocephalae Rhizoma (Baizhu) 20g, Cinnamomi Ramulus (Guizhi) 10g, Pinelliae Rhizoma (Banxia) 15g, Amomi Fructus (Sharen) 6g, Gastrodiae Rhizoma (Tianma) 10g, Glycyrrhizae Radix et Rhizoma (Gancao) 9g. | Not reported | Decocted in water to 300 mL and taken twice daily (morning and evening). |
| Wang DD 2019 | Dingxuan Granule | Gentianae Radix et Rhizoma (Longdancao) 10g, Curcumae Radix (Yujin) 10g, Citri Reticulatae Pericarpium (Chenpi) 10g, Chrysanthemi Flos (Juhua) 10g, Gastrodiae Rhizoma (Tianma) 10g, Acori Tatarinowii Rhizoma (Shichangpu) 15g, Pinelliae Rhizoma (Banxia) 15g, Atractylodis Macrocephalae Rhizoma (Baizhu) 15g, Arisaema cum Bile (Dannanxing) 15g, Poria (Fuling) 15g, Puerariae Radix (Gegen) 15g, Rhizoma Alismatis (Zexie) 15g. | Not reported | One dose daily, dissolved in boiling water,taken twice daily (morning and evening). |
| Wang HG 2016 | Xiaqi Decoction | Pinelliae Rhizoma (Banxia) 9g, Citri Grandis Exocarpium (Juhong) 9g, Armeniacae Semen Amarum (Kuxingren) 9g, Poria (Fuling) 9g, Glycyrrhizae Radix et Rhizoma (Gancao) 6g, Moutan Cortex (Mudanpi) 9g, Paeoniae Radix Alba (Baishao) 12g, Polygoni Multiflori Radix Preparata (Heshouwu) 20g. | Not reported | One dose daily, decocted in water. |
| Wang XH 2024 | Guizhi Plus Gegen Decoction combined with Danggui Shaoyao Powder | Puerariae Radix (Gegen) 30g, Cinnamomi Ramulus (Guizhi) 15g, Poria (Fuling) 20g, Rhizoma Alismatis (Zexie) 30g, Atractylodis Macrocephalae Rhizoma (Baizhu) 20g, Angelica sinensis (Oliv.) Diels (Danggui) 10g, Paeoniae Radix Alba (Baishao) 20g, Chuanxiong Rhizoma (Chuanxiong) 20g, Glycyrrhizae Radix et Rhizoma (Gancao) 3g, Zingiberis Rhizoma (Ganjiang) 5g, Jujubae Fructus (Dazao) 10g. | Centrally decocted by the Preparation Room of Wuxi Hospital Affiliated to Nanjing University of Chinese Medicine. | One dose daily, decocted and taken twice daily, 200 mL per time. |

**Supplementary Table 3** Specific ingredients and dosages of SSPR prescriptions (Continued).

| **Study ID** | **Name of prescriptions** | **Compositions** | **Preparation and source** | **Administration and dosage** |
| --- | --- | --- | --- | --- |
| Wang ZE 2022 | Dingxuan Decoction | Pinelliae Rhizoma (Banxia) 10g, Gastrodiae Rhizoma (Tianma) 15g, Atractylodis Macrocephalae Rhizoma (Baizhu) 15g, Poria cum Radix Pini (Fushen) 15g, Citri Reticulatae Pericarpium (Chenpi) 10g, Angelica sinensis (Oliv.) Diels (Danggui) 20g, Chuanxiong Rhizoma (Chuanxiong) 10gRhizoma Alismatis (Zexie) 20g, Paeoniae Radix Alba (Baishao) 15g, Uncariae Ramulus cum Uncis (Gouteng) 15g, Polygoni Multiflori Caulis (Shouwuteng) 30g, Glycyrrhizae Radix et Rhizoma (Gancao) 6g. | Uniformly supplied and decocted using automatic decoction machines by the Pharmacy Department of East Campus, Gansu Provincial Hospital of TCM. | One dose daily, taken twice daily (morning and evening), 150 mL per time. |
| Zhang DQ 2020 | Modified BBTD | Gastrodiae Rhizoma (Tianma) 15g, Pinelliae Rhizoma (Banxia) 10g, Atractylodis Macrocephalae Rhizoma (Baizhu) 12g, Poria (Fuling) 12g, Citri Reticulatae Pericarpium (Chenpi) 12g, Citri Sarcodactylis Fructus (Foshou) 12g, Chuanxiong Rhizoma (Chuanxiong) 12g, Polygalae Radix (Yuanzhi) 12g, Gardeniae Fructus (Zhizi) 10g, Albiziae Cortex (Hehuanpi) 20g, Glycyrrhizae Radix et Rhizoma (Gancao) 10g. | Centrally decocted by the Preparation Room, Heze Chinese Medicine Hospital (200 mL × 2 per dose). | One dose daily, taken twicw daily (post-meal at morning and evening) |
| Zhang JW 2019 | Added BBTD | Pinelliae Rhizoma (Banxia) 10g, Gastrodiae Rhizoma (Tianma) 10g, Atractylodis Macrocephalae Rhizoma (Baizhu) 10g, Poria (Fuling) 15g, Citri Grandis Exocarpium (Juhong) 10g, Astragali Radix (Huangqi) 15g, Rhizoma Alismatis (Zexie) 10g, Angelica sinensis (Oliv.) Diels (Danggui) 10g, Chuanxiong Rhizoma (Chuanxiong) 10g, Glycyrrhizae Radix et Rhizoma (Gancao) 6g, Zingiberis Rhizoma Recens (Shengjiang) 10g, Jujubae Fructus (Dazao) 10g. | Sourced from and decocted by the Pharmacy Department, Hubei Provincial Hospital of TCM. Concentratedly decocted to 200 mL. | Taken twice daily (morning and evening), 100 mL per time. |
| Zhou JJ 2022 | Dingxuan Decoction | Pinelliae Rhizoma (Banxia) 10g, Atractylodis Macrocephalae Rhizoma (Baizhu) 15g, Gastrodiae Rhizoma (Tianma) 15g, Poria (Fuling) 15g, Rhizoma Alismatis (Zexie) 10g, Acori Tatarinowii Rhizoma (Shichangpu) 10g, Polygoni Multiflori Caulis (Shouwuteng) 15g, Polygalae Radix (Yuanzhi) 10g, Albiziae Cortex (Hehuanpi) 15g, Achyranthis Bidentatae Radix (Niuxi) 10g, Glycyrrhizae Radix et Rhizoma (Gancao) 6g. | Sourced by hospital procurement and centrally decocted by the TCM Decoction Room of the First Affiliated Hospital of Guangxi University of Chinese Medicine. | One dose (400 mL) daily, taken warm twice daily, (30 min post-meal in the morning and evening), 200 mL per time. |

**Supplementary Table 3** Specific ingredients and dosages of SSPR prescriptions (Continued).

| **Study ID** | **Name of prescriptions** | **Compositions** | Preparation and source | Administration and dosage |
| --- | --- | --- | --- | --- |
| **SSPR therapy plus SOC combination versus SOC** | | |  |  |
| Cheng JH 2022 | Added Yuzhu Dingxuan Decoction | Poria (Fuling) 30g, Pinelliae Rhizoma (Banxia) 12g, Citri Reticulatae Pericarpium (Chenpi) 12g, Coicis Semen (Yiyiren) 30g, Rhizoma Alismatis (Zexie) 20g, Achyranthis Bidentatae Radix (Niuxi) 12g, Corni Fructus (Shanzhuyu) 25g, Ostreae Concha (Muli) 20g, Descurainiae et Lepidii Semen (Tinglizi) 20g, Glycyrrhizae Radix et Rhizoma (Gancao) 6g, Zingiberis Rhizoma Recens (Shengjiang) 6g, Jujubae Fructus (Dazao) 10g, Bambusae Caulis in Taeniam (Zhuru) 10g, Aurantii Fructus Immaturus (Zhishi) 10g, Atractylodis Rhizoma (Cangzhu) 15g. | Sourced from Henan Provincial Hospital of TCM (produced by Sichuan New Green Pharmaceutical Technology Development Co., Ltd., complying with Chinese Pharmacopoeia standards). | One dose daily, dissolved in 200 mL boiling water per dose, taken warm twice daily (after lunch and dinner). |
| Ding WJ 2019 | BBTD | Pinelliae Rhizoma (Banxia) 12g, Atractylodis Macrocephalae Rhizoma (Baizhu) 12g, Gastrodiae Rhizoma (Tianma) 10g, Poria (Fuling) 10g, Citri Reticulatae Pericarpium (Chenpi) 10g, Glycyrrhizae Radix et Rhizoma (Gancao) 3g, Zingiberis Rhizoma Recens (Shengjiang) 1 slice, Jujubae Fructus (Dazao) 2 pieces. | Decocted in water to 300 mL. | One dose daily, taken twice daily. |
| Gong XH 2024 | Added Jiawei Linggui Zhugan Decoction | Poria (Fuling) 15g, Cinnamomi Ramulus (Guizhi) 9g, Atractylodis Macrocephalae Rhizoma (Baizhu) 9g, Atractylodis Rhizoma (Cangzhu) 9g, Glycyrrhizae Radix et Rhizoma (Gancao) 6g, Rhizoma Alismatis (Zexie) 12g, Pinelliae Rhizoma (Banxia) 12g, Gastrodiae Rhizoma (Tianma) 9g, Citri Grandis Exocarpium (Juhong) 9g, Zingiberis Rhizoma Recens (Shengjiang) 6g, Jujubae Fructus (Dazao) 9g, Codonopsis Radix（Dangshen) 9g, Puerariae Radix (Gegen) 12g, Acori Tatarinowii Rhizoma (Shichangpu) 9g. | Centrally decocted by the Pharmacy Department of the TCM of Wuhan Hospital of Integrated Traditional Chinese and Western Medicine. | One dose daily (150 mL per dose), taken twice daily (post-meal in the morning and evening). |
| Guo MZ 2025 | Modified Dingxuan Zhiyun Decoction | Gastrodiae Rhizoma (Tianma) 12 g, Polygalae Radix (Yuanzhi) 6 g, Pinelliae Rhizoma (Banxia) 10g, Citri Reticulatae Pericarpium (Chenpi) 6g, Atractylodis Macrocephalae Rhizoma (Baizhu) 10g, Puerariae Radix (Gegen) 12g, Chuanxiong Rhizoma (Chuanxiong) 6g, Chrysanthemi Flos (Juhua) 10g, Trionycis Carapax (Biejia) 12g. | Not reported | One dose daily, decocted in water and taken twice daily (morning and evening). |

**Supplementary Table 3** Specific ingredients and dosages of SSPR prescriptions (Continued).

| **Study ID** | **Name of prescriptions** | **Compositions** | **Preparation and source** | **Administration and dosage** |
| --- | --- | --- | --- | --- |
| Li XQ 2018 | Xuanyun Fomula | Atractylodis Macrocephalae Rhizoma (Baizhu) 10g, Rhizoma Alismatis (Zexie) 25g, Gastrodiae Rhizoma (Tianma) 10g, Pinelliae Rhizoma (Banxia) 10g, Uncariae Ramulus cum Uncis (Gouteng) 12g, Citri Reticulatae Pericarpium (Chenpi) 10g. | Not reported | Soaked in 400 mL water for 30 min and decocted to about 200 mL; re-decocted with 300 mL water to about 150 mL; mixed filtrate and taken warm twice daily (post-meal in the morning and evening). |
| Qiu YJ 2024 | Modified Wendan Decoction | Pinelliae Rhizoma (Banxia) 9g, Poria (Fuling) 15g, Glycyrrhizae Radix et Rhizoma (Gancao) 6g, Aurantii Fructus Immaturus (Zhishi) 10g, Bambusae Caulis in Taeniam (Zhuru) 10g, Citri Grandis Exocarpium (Juhong) 10g, Zingiberis Rhizoma Recens (Shengjiang) 10g, Jujubae Fructus (Dazao) 10g. | Not reported | One dose daily, taken twice daily (morning and evening). |
| Tong XP 2024 | Jieyu Huatan Decoction + BHT-M + Flupentixol/Melitracen | Bupleuri Radix (Chaihu) 10g, Poria (Fuling) 15g, Atractylodis Macrocephalae Rhizoma (Baizhu) 10g, Angelica sinensis (Oliv.) Diels (Danggui) 10g, Paeoniae Radix Alba (Baishao) 10g, Scutellariae Radix (Huangqin) 10g, Pinelliae Rhizoma (Banxia) 10g, Citri Reticulatae Pericarpium (Chenpi) 12g, Rhizoma Alismatis (Zexie) 10g, Gastrodiae Rhizoma (Tianma) 10g, Viticis Fructus (Manjingzi) 10g, Glycyrrhizae Radix et Rhizoma (Gancao) 5g. | Sourced from the Pharmacy Department of Zhangzhou Chinese Medicine Hospital (Pien Tze Huang Pharmaceutical Co., Ltd.), meeting authentic and standardized criteria; centrally decocted to 300 mL, divided equally into two 150 mL vacuum-sealed bags. | Taken warm twice daily, 1 bag per time (30 min post-meal in the morning and evening). |

**Supplementary Table 3** Specific ingredients and dosages of SSPR prescriptions (Continued).

| **Study ID** | **Name of prescriptions** | **Compositions** | **Preparation and source** | **Administration and dosage** |
| --- | --- | --- | --- | --- |
| Wang B 2022 | BBTD | Pinelliae Rhizoma (Banxia) 10g, Gastrodiae Rhizoma (Tianma) 10g, Atractylodis Macrocephalae Rhizoma (Baizhu) 10g, Poria (Fuling) 10g, Citri Grandis Exocarpium (Juhong) 10g, Glycyrrhizae Radix et Rhizoma (Gancao) 6g, Zingiberis Rhizoma Recens (Shengjiang) 10g, Jujubae Fructus (Dazao) 10g. | Not reported | Decocted in water to 200 mL and taken warm twice daily (100 mL per time in the morning and evening). |
| Wang R 2024 | Added BBTD | Gastrodiae Rhizoma (Tianma) 10g, Pinelliae Rhizoma (Banxia) 9g, Atractylodis Macrocephalae Rhizoma (Baizhu) 20g, Poria (Fuling) 10g, Citri Reticulatae Pericarpium (Chenpi) 12g, Citri Grandis Exocarpium (Juhong) 6g, Glycyrrhizae Radix et Rhizoma (Gancao) 6g, Zingiberis Rhizoma Recens (Shengjiang) 2 slices, Jujubae Fructus (Dazao) 3 pieces. | Not reported | One dose daily, decocted in water to 300 mL (strained) and taken warm twice daily (morning and evening). |
| Xu Z 2024 | Buzhong Yiqi Decoction combined with Erchen Decoction | Astragali Radix (Huangqi) 15g, Codonopsis Radix（Dangshen) 15g, Pinelliae Rhizoma (Banxia) 10g, Citri Grandis Exocarpium (Juhong) 6g, Poria (Fuling) 9g, Atractylodis Macrocephalae Rhizoma (Baizhu) 9g, Cimicifugae Rhizoma (Shengma) 6g, Bupleuri Radix (Chaihu) 6g, Gastrodiae Rhizoma (Tianma) 3g, Glycyrrhizae Radix et Rhizoma (Gancao) 9g. | Not reported | One dose daily, decocted in water and taken twice daily (30 min post-meal). |
| Yu M 2024 | Added Wendan Decoction + Conventional WM | Pinelliae Rhizoma (Banxia) 10g, Arisaema cum Bile (Dannanxing) 15g, Aurantii Fructus Immaturus (Zhishi) 10g, Gastrodiae Rhizoma (Tianma) 10g, Atractylodis Macrocephalae Rhizoma (Baizhu) 10g, Chuanxiong Rhizoma (Chuanxiong) 10g, Acori Tatarinowii Rhizoma (Shichangpu) 10g, Curcumae Radix (Yujin) 10g, Poria cum Radix Pini (Fushen) 10g, Polygalae Radix (Yuanzhi) 6g, Rhizoma Alismatis (Zexie) 10g. | Centrally decocted and provided in vacuum-sealed bags by the Pharmacy Department of Wuhan Hospital of TCM. | One dose daily, taken twice daily (200 mL per time in the morning and evening). |

**Supplementary Table 3** Specific ingredients and dosages of SSPR prescriptions (Continued).

| **Study ID** | **Name of prescriptions** | **Compositions** | **Preparation and source** | **Administration and dosage** |
| --- | --- | --- | --- | --- |
| Zeng YS 2024 | Modified BBTD + VRT | Pinelliae Rhizoma (Banxia) 9g, Atractylodis Macrocephalae Rhizoma (Baizhu) 10g, Citri Reticulatae Pericarpium (Chenpi) 6g, Poria (Fuling) 12g, Gastrodiae Rhizoma (Tianma) 9g, Zingiberis Rhizoma Recens (Shengjiang) 6g, Jujubae Fructus (Dazao) 9g, Rhizoma Alismatis (Zexie) 6g, Coicis Semen (Yiyiren) 12g, Glycyrrhizae Radix et Rhizoma (Gancao) 6g. | Centrally decocted by the Pharmacy Department of The Second People's Hospital Affiliated to Fujian University of TCM (Specification: 200 mL/bag, 2 bags/day). | One dose daily, taken warm 30 min post-meal in the morning and evening). |
| Zhang XX 2024 | Added BBTD + BHT-M | Pinelliae Rhizoma (Banxia) 10g, Atractylodis Macrocephalae Rhizoma (Baizhu) 15g, Gastrodiae Rhizoma (Tianma) 12g, Poria (Fuling) 15g, Rhizoma Alismatis (Zexie) 10g, Citri Reticulatae Pericarpium (Chenpi) 10g, Zingiberis Rhizoma Recens (Shengjiang) 6g, Jujubae Fructus (Dazao) 10g, Glycyrrhizae Radix et Rhizoma (Gancao) 6g, Margarita Pulverata (Zhenzhufen) 30g, Tribuli Fructus (Jili) 15g, Uncariae Ramulus cum Uncis (Gouteng) 15g. | Not reported | One dose daily, decocted in water and taken twice daily. |
| Zhang Y 2023 | Xuanfu Daizhe Decoction + BHT-M | Inulae Flos (Xuanfuhua) 12g, Ginseng Radix et Rhizoma (Renshen) 12g, Zingiberis Rhizoma Recens (Shengjiang) 8g, Haematitum (Daizheshi) 6g, Glycyrrhizae Radix et Rhizoma (Gancao) 6g, Pinelliae Rhizoma (Banxia) 8g, Glycyrrhizae Radix et Rhizoma (Gancao) 6g, Jujubae Fructus (Dazao) 4 pieces. | Not reported | One dose daily, decocted in water and taken twice daily (post-meal). |
| Zhou YJ 2025 | Huatan Pingxuan Fomula + BHT-M | Pinelliae Rhizoma (Banxia) 12g, Gastrodiae Rhizoma (Tianma) 15g, Atractylodis Macrocephalae Rhizoma (Baizhu) 15g, Codonopsis Radix（Dangshen) 20g, Astragali Radix (Huangqi) 30g, Citri Grandis Exocarpium (Juhong) 15g, Phellodendri Chinensis Cortex (Huangbo) 10g, Zingiberis Rhizoma (Ganjiang) 15g, Poria (Fuling) 30g, Rhizoma Alismatis (Zexie) 10g, Atractylodis Rhizoma (Cangzhu) 15g, Hordei Fructus Germinatus (Maiya) 15g. | Not reported | One dose daily, decocted in water to 300 mL (1 dose) and taken in 3 divided portions (morning, afternoon, and evening). |

**Note:** BBTD, Banxia Baizhu Tianma Decoction; BHT-M, Betahistine Mesylate; SOC, Standard of care; SSPR, Spleen-strengthening and phlegm-resolving; TCM, Traditional Chinese medicine; VRT, Vestibular rehabilitation therapy; WM, Western medicine.

**Supplementary Table 4** Overall risk of bias for each study and each outcome.

| **Study ID** | **DHI** | **Dizziness resolution rate** | **VAS** | **TCM syndrome score** | **Clinical effective rate** | **Recurrence rate** |
| --- | --- | --- | --- | --- | --- | --- |
| **SSPR therapy versus no intervention** | | | | | | |
| Fan SH 2020 | Some concerns | Some concerns | / | Some concerns | Some concerns | High risk |
| Liu J 2022 | Some concerns | Some concerns | / | Some concerns | Some concerns | / |
| Shu F 2022 | Some concerns | / | Some concerns | / | / | / |
| Zhang Y et al2021 | Some concerns | High risk | / | / | / | / |
| **SSPR therapy versus placebo** | | | | | | |
| Liu S et al2025a | Some concerns | Some concerns | Some concerns | Some concerns | Some concerns | / |
| Wu WL 2023 | High risk | / | / | High risk | High risk | / |
| Zhu YJ 2021 | High risk | High risk | / | High risk | High risk | / |
| **SSPR therapy versus SOC** | | | | | | |
| Cai HC et al2016 | Some concerns | / | / | Some concerns | / | / |
| Huang SH 2020 | High risk | High risk | / | High risk | High risk | / |
| Huang ST et al2021 | Some concerns | High risk | Some concerns | / | / | / |
| Huang Y et al2017 | Some concerns | / | / | / | / | / |
| Lin XQ 2019 | Some concerns | / | Some concerns | Some concerns | Some concerns | / |
| Liu YF et al2026 | Some concerns | / | Some concerns | Some concerns | Some concerns | / |
| Qin ZG 2020 | / | High risk | / | / | / | / |
| Shao DF et al2023 | Some concerns | / | / | / | / | / |
| Wang DD et al2019a | / | / | / | Some concerns | / | / |
| Wang DD et al2019b | / | / | / | Some concerns | / | / |
| Wang HG et al2016a | Some concerns | / | / | Some concerns | / | / |
| Wang XH 2024 | High risk | High risk | High risk | High risk | Some concerns | / |
| Wang ZE 2022 | Some concerns | / | Some concerns | Some concerns | Some concerns | / |

**Supplementary Table 4** Overall risk of bias for each study and each outcome (Continued).

| **Study ID** | **DHI** | **Dizziness resolution rate** | **VAS** | **TCM syndrome score** | **Clinical effective rate** | **Recurrence rate** |
| --- | --- | --- | --- | --- | --- | --- |
| **SSPR therapy versus SOC** | | | | | | |
| Zhang DQ 2020 | Some concerns | / | / | / | Some concerns | / |
| Zhang JW 2019 | Some concerns | / | Some concerns | Some concerns | Some concerns | / |
| Zhou JJ 2022 | High risk | / | High risk | High risk | High risk | / |
| **SSPR therapy plus SOC combination versus SOC** | | | | | | |
| Cheng JH 2022 | High risk | High risk | / | High risk | High risk | / |
| Ding WJ et al2019 | Some concerns | / | / | / | / | / |
| Gong XH 2024 | High risk | / | / | High risk | High risk | High risk |
| Guo MZ et al2025 | Some concerns | / | / | / | / | High risk |
| Li XQ et al2018 | High risk | High risk | / | / | / | High risk |
| Liu S et al2025b | Some concerns | Some concerns | Some concerns | Some concerns | Some concerns | / |
| Qiu YJ et al2024 | High risk | / | / | High risk | High risk | High risk |
| Tong XP 2024 | High risk | / | / | High risk | High risk | / |
| Wang B et al2022 | Some concerns | / | / | Some concerns | Some concerns | / |
| Wang HG et al2016b | Some concerns | / | / | Some concerns | / | / |
| Wang R et al2024 | Some concerns | Some concerns | / | / | Some concerns | / |
| Xu Z et al2024 | / | / | / | Some concerns | Some concerns | / |
| Yu M 2024 | Some concerns | Some concerns | Some concerns | Some concerns | Some concerns | High risk |
| Zeng YS et al2024 | Some concerns | / | / | Some concerns | / | / |
| Zhang XX 2024 | Some concerns | / | / | Some concerns | / | / |
| Zhang Y et al2023 | High risk | High risk | / | / | / | High risk |
| Zhou YJ et al2025 | Some concerns | / | Some concerns | / | Some concerns | / |

Note: DHI, Dizziness handicap inventory; SOC, Standard of care; SSPR, Spleen-strengthening and phlegm-resolving; TCM, Traditional Chinese Medicine; VAS, Visual analogue scale.

**Supplementary Table 5** Recurrence rate.

| Study | Experimental group | | Control group | | Follow-up period | Indicators |
| --- | --- | --- | --- | --- | --- | --- |
|  | Recurrence, n(%) | Sample size | Recurrence, n(%) | Sample size |  |  |
| **SSPR therapy versus no intervention** | | | | | | |
| Fan SH 2020 | 1(3.33%) | 30 | 8(26.67%) | 30 | 3 months | BPPV |
| Subtotal | 1(3.33%) | 30 | 8(26.67%) | 30 |  |  |
| **SSPR therapy plus SOC combination versus SOC** | | | | | | |
| 6-month follow-up period (RR = 0.35, 95%CI: [0.15, 0.78], I^2^ = 0%, *p =* 0.01) | | | | | | |
| Guo MZ 2025 | 2 (5.13%) | 39 | 8 (20.51%) | 39 | 6 months | RD/BPPV* |
| Li XQ 2018 | 2 (3.85%) | 52 | 3 (5.66%) | 53 | 6 months | BPPV |
| Qiu YJ 2024 | 3 (8.33%) | 36 | 7 (19.44%) | 36 | 6 months | BPPV |
| Zhang Y 2023^a^ | 0 (0.00%) | 38 | 7 (17.95%) | 39 | 6 months | BPPV |
| Subtotal | 7 (4.24%) | 165 | 25 (14.97%) | 167 |  |  |
| Other follow-up periods | | | | | | |
| Gong XH 2024 | 3 (8.82%) | 34 | 8 (25.00%) | 32 | 14 days | RD |
| Yu M 2024^a^ | 1 (3.33%) | 30 | 9 (30.00%) | 30 | 1 month | RD |
| Yu M 2024^b^ | 2 (6.67%) | 30 | 12 (40.00%) | 30 | 3 months | RD |
| Zhang Y 2023^b^ | 0 (0.00%) | 38 | 4 (10.26%) | 39 | 1 month | BPPV |
| Zhang Y 2023^c^ | 1 (2.63%) | 38 | 9 (23.08%) | 39 | 12 months | BPPV |

**Note:** BPPV, Benign paroxysmal positional vertigo; CI, Confidence interval; n, number of recurrent cases; RD, Residual dizziness; RR, Relative risk; SOC, Standard of care; SSPR, Spleen-strengthening and phlegm-resolving.

* Recurrence in this study was not explicitly specified as BPPV or RD.

**Supplementary Table 6** Summary of effect estimations.

| **Outcomes** | **N, n** | **Estimate effect, 95% CI** | ***P*** |
| --- | --- | --- | --- |
| **SSPR therapy versus no intervention** | | | |
| DHI | N = 4, n = 411 | MD = -6.95, 95%CI: [-13.52, -0.37], I^2^ = 96% | p = 0.04^b^ |
| Dizziness resolution rate | N = 3, n = 350 | RR = 1.18, 95%CI: [0.85, 1.65], I^2^ = 44% | p = 0.31^a^ |
| VAS | N = 1, n = 61 | MD = -1.00, 95%CI: [-1.78, -0.22] | p = 0.01^b^ |
| TCM syndrome score | N = 2, n = 260 | SMD = -0.80, 95%CI: [-2.25, 0.65], I^2^ = 95% | *p* = 0.28^a^ |
| Clinical effective rate | N = 2, n = 260 | RR = 1.06, 95%CI: [0.90, 1.24], I^2^ = 71% | *p* = 0.50^a^ |
| Recurrence rate | N = 1, n = 60 | RR = 0.13, 95%CI: [0.02, 0.94] | *p* = 0.04^b^ |
| **SSPR therapy versus placebo** | | | |
| DHI | N = 3, n = 185 | MD = -10.57, 95%CI: [-14.89, -6.25], I^2^ = 72% | p < 0.00001^c^ |
| Dizziness resolution rate | N = 2, n = 111 | RR = 3.49, 95%CI: [0.28, 43.69], I^2^ = 70% | p = 0.33^a^ |
| VAS | N = 1, n = 56 | MD = -0.93, 95%CI: [-1.76, -0.10] | p = 0.03^b^ |
| TCM syndrome score | N = 3, n = 185 | SMD = -1.19, 95%CI: [-1.74, -0.64], I^2^ = 67% | p < 0.0001^c^ |
| Clinical effective rate | N = 3, n = 185 | RR = 1.40, 95%CI: [1.11, 1.78], I^2^ = 51% | p = 0.005^c^ |
| **SSPR therapy versus SOC** | | | |
| DHI | N = 13, n = 909 | MD = -9.24, 95%CI: [-10.98, -7.50], I^2^ = 81% | p < 0.00001^c^ |
| Dizziness resolution rate | N = 4, n = 276 | RR = 1.56, 95%CI: [1.15, 2.11], I^2^ = 0% | p = 0.004^c^ |
| VAS | N = 7, n = 474 | MD = -1.32, 95%CI: [-1.71, -0.93], I^2^ = 47% | p < 0.00001^c^ |
| TCM syndrome score | N = 11, n = 770 | SMD = -1.84, 95%CI: [-2.49, -1.20], I^2^ = 93% | p < 0.00001^c^ |
| Clinical effective rate | N = 8, n = 547 | RR = 1.19, 95%CI: [1.12, 1.28], I^2^ = 0% | p < 0.00001^c^ |
| **SSPR therapy plus SOC combination versus SOC** | | | |
| DHI | N = 16, n = 1151 | MD = -9.33, 95%CI: [-11.45, -7.21], I^2^ = 96% | p < 0.00001^c^ |
| Dizziness resolution rate | N = 6, n = 440 | RR = 1.66, 95%CI: [1.32, 2.07], I^2^ = 0% | p < 0.0001^c^ |
| VAS | N = 3, n = 176 | MD = -1.39, 95%CI: [-2.20, -0.58], I^2^ = 79% | p = 0.0008^c^ |
| TCM syndrome score | N = 11, n = 737 | SMD = -1.39, 95%CI: [-1.97, -0.81], I^2^ = 92% | p < 0.00001^c^ |
| Clinical effective rate | N = 10, n = 627 | RR = 1.18, 95%CI: [1.11, 1.25], I^2^ = 0% | *p* < 0.00001^c^ |
| Recurrence rate | N = 4, n = 332 | RR = 0.35, 95%CI: [0.15, 0.78], I^2^ = 0% | *p =* 0.01^b^ |

**Note:** ^a^ No statistically significant difference compared with the control group (p *>* 0.05); ^b^ Statistically significant difference compared with the control group (p *≥* 0.01, p *<* 0.05); ^c^ Highly statistically significant difference compared with the control group (p *<* 0.01), with the experimental group significantly superior to the control group. BPPV, Benign paroxysmal positional vertigo; CI, Confidence interval; DHI, Dizziness handicap inventory; MD, Mean difference; N, number of included trials; n, number of participants; RD, Residual dizziness; RR, Relative risk; SMD, Standardized mean difference; SOC, Standard of care; SSPR, Spleen-strengthening and phlegm-resolving; TCM, Traditional Chinese Medicine; VAS, Visual analogue scale; VRT, Vestibular rehabilitation therapy; WM, Western medicine.

**Supplementary Table 7** Subgroup analysis results.

| **Outcomes** | **Subgroup variables** | **Subgroup Categories** | **N, n** | **Estimate effect, 95% CI** | ***P_heterogeneity_*** | ***P_interaction_*** |
| --- | --- | --- | --- | --- | --- | --- |
| **SSPR therapy versus SOC** | | | | | | |
| DHI | SOC type | Vestibular monotherapy | N = 8, n = 530 | MD = -8.83, 95%CI:[-10.84, -6.82], I^2^ = 74% | *p* < 0.00001^c^ | */* |
|  |  | Central psychotropic-containing regimen | N = 4, n = 299 | MD = -11.35, 95%CI: [-14.05, -8.64], I^2^ = 73% | *p* < 0.00001^c^ |  |
|  |  | Standardized phytopharmaceutical monotherapy | N = 1, n = 80 | MD = -5.56, 95%CI: [-7.71, -3.41] | *p* < 0.00001^c^ |  |
|  | Number of herbal components | < 10 | N = 4, n = 320 | MD = -10.82, 95%CI:[-14.65, -6.99], I^2^ = 91% | *p* < 0.00001^c^ | *p* = 0.27^a^ |
|  |  | ≥ 10 | N = 9, n = 589 | MD = -8.46, 95%CI:[-10.12, -6.79], I^2^ = 64% | *p* < 0.00001^c^ |  |
|  | Treatment duration | ≤ 14 days | N = 11, n = 791 | MD = -9.49, 95%CI:[-11.37, -7.62], I^2^ = 83% | *p* < 0.00001^c^ | *p* = 0.14^a^ |
|  |  | > 14 days | N = 2, n = 118 | MD = -6.84, 95%CI:[-9.80, -3.87], I^2^ = 0% | *p* < 0.00001^c^ |  |
| TCM syndrome score | SOC type | Vestibular monotherapy | N = 8, n = 530 | SMD = -1.37, 95%CI: [-1.98, -0.77], I^2^ = 90% | *p* < 0.00001^c^ | */* |
|  |  | Central psychotropic-containing regimen | N = 2, n = 180 | SMD = -2.62, 95%CI: [-3.84, -1.39], I^2^ = 86% | *p* < 0.0001^c^ |  |
|  |  | VRT | N = 1, n = 60 | SMD = -4.03, 95%CI: [-4.95, -3.11] | *p* < 0.00001^c^ |  |
|  | Number of herbal components | < 10 | N = 2, n = 180 | SMD = -2.62, 95%CI: [-3.84, -1.39], I^2^=86% | *p* < 0.0001^c^ | *p* = 0.19^a^ |
|  |  | ≥ 10 | N = 9, n = 590 | SMD = -1.66, 95%CI: [-2.36, -0.97], I^2^=93% | *p* < 0.00001^c^ |  |

**Supplementary Table 7** Subgroup analysis results (Continued).

| **Outcomes** | **Subgroup variables** | **Subgroup Categories** | **N, n** | **Estimate effect, 95% CI** | ***P_heterogeneity_*** | ***P_interaction_*** |
| --- | --- | --- | --- | --- | --- | --- |
| **SSPR therapy plus SOC combination versus SOC** | | | | | | |
| DHI | SOC type | WM | N = 13, n = 957 | MD = -8.77, 95%CI: [-10.86, -6.68], I^2^ = 96% | *p* < 0.00001^c^ | */* |
|  |  | VRT | N = 2, n = 116 | MD = -7.88, 95%CI: [-9.52, -6.25], I^2^ = 4% | *p* < 0.00001^c^ |  |
|  |  | VRT plus WM | N = 1, n = 78 | MD = -20.10, 95%CI: [-22.12, -18.08] | *p* < 0.00001^c^ |  |
|  | Number of herbal components | < 10 | N = 9, n = 710 | MD = -10.04, 95%CI:[-13.54, -6.54], I^2^ = 98% | *p* < 0.00001^c^ | *p* = 0.38^a^ |
|  |  | ≥ 10 | N = 7, n = 441 | MD = -8.41, 95%CI:[-9.49, -7.33], I^2^ = 50% | *p* < 0.00001^c^ |  |
|  | Treatment duration | ≤ 7 days | N = 3, n = 225 | MD = -5.74, 95%CI:[-10.40, -1.07], I^2^ = 97% | *p* = 0.02^b^ | *p* = 0.18^a^ |
|  |  | 7 days to 14 days | N = 10, n = 750 | MD = -10.66, 95%CI:[-13.58, -7.73], I^2^ = 97% | *p* < 0.00001^c^ |  |
|  |  | > 14 days | N = 3, n = 176 | MD = -8.32, 95%CI:[-10.20, -6.43], I^2^ = 28% | *p* < 0.00001^c^ |  |
| TCM syndrome score | SOC type | WM | N = 9, n = 621 | SMD = -1.50, 95%CI: [-2.21, -0.80], I^2^ = 93% | *p* < 0.0001^c^ | *p* = 0.20^a^ |
|  |  | VRT | N = 2, n = 116 | SMD = -0.90, 95%CI: [-1.48, -0.33], I^2^ = 55% | *p* = 0.002^c^ |  |
|  | Number of herbal components | < 10 | N = 4, n = 308 | SMD = -2.41, 95%CI: [-3.86, -0.96], I^2^ = 96% | *p* = 0.001^c^ | *p* = 0.03^b^ |
|  |  | ≥ 10 | N = 7, n = 429 | SMD = -0.83, 95%CI: [-1.03, -0.63], I^2^ = 0% | *p* < 0.00001^c^ |  |
|  | Treatment duration | ≤ 14 days | N = 8, n = 561 | SMD = -1.59, 95%CI: [-2.39, -0.79], I^2^ = 94% | *p* < 0.0001^c^ | *p* = 0.11^a^ |
|  |  | > 14 days | N = 3, n = 176 | SMD = -0.88, 95%CI: [-1.21, -0.55], I^2^ = 11% | *p* < 0.00001^c^ |  |

**Note:** ^a^ No statistically significant difference compared with the control group (*p >* 0.05); ^b^ Statistically significant difference compared with the control group (*p ≥* 0.01, *p <* 0.05); ^c^ Highly statistically significant difference compared with the control group (*p <* 0.01), with the experimental group significantly superior to the control group. CI, Confidence interval; DHI, Dizziness handicap inventory; MD, Mean difference; N, number of included trials; n, number of participants; SMD, Standardized mean difference; SOC, Standard of care; SSPR, Spleen-strengthening and phlegm-resolving; TCM, Traditional Chinese Medicine; VRT, Vestibular rehabilitation therapy; WM, Western medicine.

**Supplementary Table 8** GRADE evidence profiles.

| **Certainty assessment** | | | | | | | **Number of patients** | | **Effect Estimates (95%CI)** | **Certainty** | **Quality** |
| --- | --- | --- | --- | --- | --- | --- | --- | --- | --- | --- | --- |
| **Outcomes** | **Number of studies** | **Risk of bias** | **Inconsistency** | **Indirectness** | **Imprecision** | **Publication bias** | **Experimental** | **Control** |  |  |  |
| **SSPR therapy versus no-intervention** | | | | | | | | | | | |
| DHI | 4 | serious^a^ | serious^b^ | not serious | not serious | none | 205 | 206 | MD -6.95 (-13.52 to -0.37) | ⨁⨁◯◯ | CRITICAL |
|  |  |  |  |  |  |  |  |  |  | Low^a,b^ |  |
| dizziness resolution rate | 3 | serious^a^ | not serious | not serious | very serious^c,d^ | none | 114/175 (65.1%) | 100/175 (57.1%) | RR 1.18 (0.85 to 1.65) | ⨁◯◯◯ | CRITICAL |
|  |  |  |  |  |  |  |  |  |  | Very low^a,c,d^ |  |
| VAS | 1 | serious^a^ | not serious | not serious | serious^c^ | none | 30 | 31 | MD -1 (-1.78 to -0.22) | ⨁⨁◯◯ | IMPORTANT |
|  |  |  |  |  |  |  |  |  |  | Low^a,c^ |  |
| TCM syndrome score | 2 | serious^a^ | serious^b^ | not serious | very serious^c,d^ | none | 130 | 130 | SMD -0.8 (-2.25 to 0.65) | ⨁◯◯◯ | IMPORTANT |
|  |  |  |  |  |  |  |  |  |  | Very low^a,b,c,d^ |  |
| clinical effective rate | 2 | serious^a^ | serious^b^ | not serious | serious^c,d^ | none | 126/130 (96.9%) | 122/130 (93.8%) | RR 1.06 (0.90 to 1.24) | ⨁◯◯◯ | IMPORTANT |
|  |  |  |  |  |  |  |  |  |  | Very low^a,b,c,d^ |  |
| recurrence rate | 1 | serious^a^ | not serious | not serious | serious^c^ | none | 1/30 (3.3%) | 8/30 (26.7%) | RR 0.13 (0.02 to 0.94) | ⨁⨁◯◯ | CRITICAL |
|  |  |  |  |  |  |  |  |  |  | Low^a,c^ |  |
| **SSPR therapy versus placebo** | | | | | | | | | | | |
| DHI | 3 | serious^a^ | serious^b^ | not serious | serious^c^ | none | 93 | 92 | MD -10.57 (-14.89 to -6.25) | ⨁◯◯◯ | CRITICAL |
|  |  |  |  |  |  |  |  |  |  | Very low^a,b,c^ |  |
| dizziness resolution rate | 2 | serious^a^ | serious^b^ | not serious | very serious^c,d^ | none | 23/57 (40.4%) | 10/54 (18.5%) | RR 3.49 (0.28 to 43.69) | ⨁◯◯◯ | CRITICAL |
|  |  |  |  |  |  |  |  |  |  | Very low^a,b,c,d^ |  |
| VAS | 1 | serious^a^ | not serious | not serious | serious^c^ | none | 28 | 28 | MD -0.93 (-1.76 to -0.1) | ⨁⨁◯◯ | IMPORTANT |
|  |  |  |  |  |  |  |  |  |  | Low^a,c^ |  |
| TCM syndrome score | 3 | serious^a^ | serious^b^ | not serious | serious^c^ | none | 93 | 92 | SMD -1.19 (-1.74 to -0.64) | ⨁◯◯◯ | IMPORTANT |
|  |  |  |  |  |  |  |  |  |  | Very low^a,b,c^ |  |
| clinical effective rate | 3 | serious^a^ | serious^b^ | not serious | serious^c^ | none | 85/93 (91.4%) | 59/92 (64.1%) | RR 1.40 (1.11 to 1.78) | ⨁◯◯◯ | IMPORTANT |
|  |  |  |  |  |  |  |  |  |  | Very low^a,b,c^ |  |

**Supplementary Table 8** GRADE evidence profiles (Continued).

| **Certainty assessment** | | | | | | | **Number of patients** | | **Effect Estimates (95%CI)** | **Certainty** | **Importance** |
| --- | --- | --- | --- | --- | --- | --- | --- | --- | --- | --- | --- |
| **Outcomes** | **Number of studies** | **Risk of bias** | **Inconsistency** | **Indirectness** | **Imprecision** | **Publication bias** | **Experimental** | **Control** |  |  |  |
| **SSPR therapy versus SOC** | | | | | | | | | | | |
| DHI | 13 | serious^a^ | serious^b^ | not serious | not serious | none | 457 | 452 | MD -9.24 (-10.98 to -7.50) | ⨁⨁◯◯ | CRITICAL |
|  |  |  |  |  |  |  |  |  |  | Low^a,b^ |  |
| dizziness resolution rate | 4 | serious^a^ | not serious | not serious | serious^c^ | none | 59/138 (42.8%) | 36/138 (26.1%) | RR 1.56 (1.15 to 2.11) | ⨁⨁◯◯ | CRITICAL |
|  |  |  |  |  |  |  |  |  |  | Low^a,c^ |  |
| VAS | 7 | serious^a^ | not serious | not serious | not serious | none | 239 | 235 | MD -1.32 (-1.71 to -0.93) | ⨁⨁⨁◯ | IMPORTANT |
|  |  |  |  |  |  |  |  |  |  | Moderate^a^ |  |
| TCM syndrome score | 11 | serious^a^ | serious^b^ | not serious | not serious | publication bias strongly suspected^f^ | 367 | 403 | SMD -1.84 (-2.49 to -1.20) | ⨁◯◯◯ | IMPORTANT |
|  |  |  |  |  |  |  |  |  |  | Very low^a,b,f^ |  |
| clinical effective rate | 8 | serious^a^ | not serious | not serious | not serious | none | 261/276 (94.6%) | 212/271 (78.2%) | RR 1.19 (1.12 to 1.28) | ⨁⨁⨁◯ | IMPORTANT |
|  |  |  |  |  |  |  |  |  |  | Moderate^a^ |  |
| **SSPR therapy plus SOC combination versus SOC** | | | | | | | | | | | |
| DHI | 16 | serious^a^ | not serious | not serious | not serious | none | 577 | 574 | MD -9.33 (-11.45 to -7.21) | ⨁⨁⨁◯ | CRITICAL |
|  |  |  |  |  |  |  |  |  |  | Moderate^a^ |  |
| dizziness resolution rate | 6 | very serious^e^ | not serious | not serious | serious^c^ | none | 94/219 (42.9%) | 54/221 (24.4%) | RR 1.66 (1.32 to 2.07) | ⨁◯◯◯ | CRITICAL |
|  |  |  |  |  |  |  |  |  |  | Very low^c,e^ |  |
| VAS | 3 | serious^a^ | serious^b^ | not serious | serious^c^ | none | 88 | 88 | MD -1.39 (-2.2 to -0.58) | ⨁◯◯◯ | IMPORTANT |
|  |  |  |  |  |  |  |  |  |  | Very low^a,b,c^ |  |
| TCM syndrome score | 11 | serious^a^ | not serious | not serious | not serious | publication bias strongly suspected^f^ | 369 | 368 | SMD -1.39 (-1.97 to -0.81) | ⨁⨁◯◯ | IMPORTANT |
|  |  |  |  |  |  |  |  |  |  | Low^a,f^ |  |
| clinical effective rate | 10 | serious^a^ | not serious | not serious | not serious | publication bias strongly suspected^f^ | 297/314 (94.6%) | 246/313 (78.6%) | RR 1.18 (1.11 to 1.25) | ⨁⨁◯◯ | IMPORTANT |
|  |  |  |  |  |  |  |  |  |  | Low^a,f^ |  |

**Supplementary Table 8** GRADE evidence profiles (Continued).

| **Certainty assessment** | | | | | | | **Number of patients** | | **Effect Estimates (95%CI)** | **Certainty** | **Importance** |
| --- | --- | --- | --- | --- | --- | --- | --- | --- | --- | --- | --- |
| **Outcomes** | **Number of studies** | **Risk of bias** | **Inconsistency** | **Indirectness** | **Imprecision** | **Publication bias** | **Experimental** | **Control** |  |  |  |
| recurrence rate | 4 | very serious^e^ | not serious | not serious | serious^c^ | none | 7/165 (4.2%) | 25/167 (15.0%) | RR 0.35 (0.15 to 0.78) | ⨁◯◯◯ | CRITICAL |
|  |  |  |  |  |  |  |  |  |  | Very low^c,e^ |  |

a. Some concern risk of bias in the majority of trials

b. High heterogeneity persisted despite extensive subgroup and sensitivity analyses

c. Limited sample size

d. No significant difference between groups

e. High risk of bias in the majority of trials

f. Egger's test was significant in publication bias (p<0.05) for meta-analyses with 10 or more trials

**Note:** BPPV, Benign paroxysmal positional vertigo; CI, Confidence interval; DHI, Dizziness handicap inventory; MD, Mean difference; RD, Residual dizziness; RR, Relative risk; SMD, Standardized mean difference; SOC, Standard of care; SSPR, Spleen-strengthening and phlegm-resolving; TCM, Traditional Chinese Medicine; VAS, Visual analogue scale.

**Supplementary Table 9** Adverse events.

| **Study** | **Experimental group** | **Control group** | **Sample size** |
| --- | --- | --- | --- |
| **SSPR therapy versus no intervention** (RR = 0.52, 95%CI: [0.05, 5.40], *P* = 0.58) | | | |
| Fan SH 2020 | / | / | 30/30 |
| Liu J 2022 | / | / | 100/100 |
| Shu F 2022 | Dizziness (n = 1) | Diarrhea(n = 1), Nausea (n = 1) | 30/31 |
| **SSPR therapy versus placebo** | | | |
| Wu WL 2023 | / | / | 36/38 |
| Zhu YJ 2021 | / | / | 29/26 |
| **SSPR therapy versus SOC** (RR = 0.26, 95%CI: [0.07, 1.06], I^2^ = 0%, *P* = 0.06) | | | |
| Huang SH 2020 | / | / | 38/38 |
| Huang Y 2017 | / | Abdominal distension (n = 1) | 30/30 |
| Lin XQ 2019 | / | / | 30/30 |
| Qin ZG 2020 | Nausea (n = 1) | Headache (n = 2) | 30/30 |
| Wang DD 2019a | / | Nausea and vomiting (n = 2), Constipation (n = 1) | 40/40 |
| Wang DD 2019b | / | / | 40/40 |
| Wang HG 2016a | / | Dry mouth (n = 2), Constipation (n = 1) | 60/60 |
| Wang XH 2024 | / | / | 30/30 |
| Wang ZE 2022 | / | / | 45/44 |
| Zhang JW 2019 | / |  | 30/30 |
| Zhou JJ 2022 | / | / | 34/31 |
| **SSPR therapy plus SOC combination versus SOC** (RR = 1.22, 95%CI: [0.48, 3.09], I^2^ = 0%, *p* = 0.68) | | | |
| Cheng JH 2022 | Anorexia (n = 1) | Anorexia (n = 2) | 30/30 |
| Gong XH 2024 | Gastrointestinal symptoms (nausea, abdominal distension, and loose stools) (n = 3) | Gastrointestinal symptoms (nausea, abdominal distension, and loose stools) (n = 1) | 34/32 |

**Supplementary Table 9** Adverse events (Continued).

| **Study** | **Experimental group** | **Control group** | **Sample size** |
| --- | --- | --- | --- |
| Guo MZ 2025 | Nausea and vomiting (n = 2), Abnormal liver function (n = 1), Abnormal renal function (n = 1) | Nausea and vomiting (n = 3) | 39/39 |
| Li XQ 2018 | / | / | 52/53 |
| Qiu YJ 2024 | Mild gastrointestinal discomfort (n = 2) | Mild gastrointestinal discomfort (n = 2) | 36/36 |
| Tong XP 2024 | / | / | 31/32 |
| Wang HG 2016b | / | / | 60/60 |
| Wang R 2024 | / | / | 41/41 |
| Yu M 2024 | / | / | 30/30 |
| Zhang Y 2023***** | / | Gastric discomfort (n = 1)***** | 40/40***** |

**Note:** CI, Confidence interval; n, number of patients with the specific adverse event; RR, Relative risk; SOC, Standard of care; SSPR, Spleen-strengthening and phlegm-resolving.

***** Reported as a dropout due to an adverse event without providing a complete adverse event profile; excluded from the safety meta-analysis. For this study, the sample size represents the initial number of randomized participants in each group.

**Supplementary Figure 1** Forest plot of subgroup analyses of DHI according to the number of herbal components.

**Supplementary Figure 2** Sensitivity analysis of dizziness resolution rate in comparison of SSPR therapy plus SOC combination versus SOC.

**Supplementary Figure 3** Sensitivity analysis of TCM syndrome scores.

**Supplementary Figure 4** Funnel plots.

Note: (A) DHI (SSPR therapy versus SOC); (B) DHI (SSPR therapy plus SOC combination versus SOC); (C) TCM syndrome score (SSPR therapy versus SOC); (D) TCM syndrome score (SSPR therapy plus SOC combination versus SOC); (E) Clinical effective rate (SSPR therapy plus SOC combination versus SOC).

**Supplementary Figure 5** Egger’s test results.

Note: (A) DHI (SSPR therapy versus SOC); (B) DHI (SSPR therapy plus SOC combination versus SOC); (C) TCM syndrome score (SSPR therapy versus SOC); (D) TCM syndrome score (SSPR therapy plus SOC combination versus SOC); (E) Clinical effective rate (SSPR therapy plus SOC combination versus SOC).
